# Peripheral blood cytokines during early and post-acute stages of SARS-CoV-2 infection are associated with disease severity and long-term symptoms

**DOI:** 10.3389/fimmu.2026.1870109

**Published:** 2026-07-15

**Authors:** Calen Mendall, Xumin Li, Vivek Pakanati, Cindy Liu, Tracy Wang, Daniel Morelli, Anna Korpak, Aaron Baraff, Stuart N. Isaacs, Kyong-Mi Chang, Elizabeth Le, Mark Holodniy, Jonathan D. Sugimoto, Nicholas L. Smith, Jennifer S. Lee, Jennifer M. Ross, Javeed A. Shah

**Affiliations:** 1 VA Puget Sound Health Care System, Seattle, WA, United States; 2 Department of Epidemiology, University of Washington, Seattle, WA, United States; 3 Corporal Michael J. Crescenz VA Medical Center, Philadelphia, PA, United States; 4 Perelman School of Medicine, University of Pennsylvania, Philadelphia, PA, United States; 5 VA Palo Alto Health Care System, Palo Alto, CA, United States; 6 International Vaccine Institute, Seoul, Republic of Korea; 7 Division of Endocrinology, Gerontology, and Metabolism, Stanford University, Palo Alto, CA, United States; 8 Division of Allergy and Infectious Diseases, University of Washington, Seattle, WA, United States

**Keywords:** COVID-19, cytokines, long Covid, SARS-CoV-2, U.S. veterans

## Abstract

**Background:**

U.S. Veterans experience a high burden of COVID-19; characterizing immune responses associated with COVID-19 outcomes could help improve treatment. Changes in peripheral blood cytokines over time may predict both acute outcomes and long COVID symptoms.

**Methods:**

Cytokine concentrations were quantified from peripheral blood collected 0–7 days (early) and 14–42 days (post-acute) after enrollment from SARS-CoV-2 positive participants in the EPIC^3^ study, a prospective, longitudinal cohort following U.S. Veterans. Responses were correlated with Veterans Affairs Severity Index for COVID-19 criteria and chronic symptoms with the modified Medical Research Council Dyspnea scale, Patient-Reported Outcomes Measurement Information System (PROMIS) Cognitive function, and PROMIS Fatigue scores 3 months after enrollment (60–135 days). Trends in cytokine concentration with COVID-19 severity were assessed. Odds of COVID-19 severity and long-term symptoms were estimated with logistic regression adjusted for sex, age, and morbidity. Longitudinal changes in cytokine concentration were examined for participants sampled during both time periods, by severity and long-term symptom group.

**Results:**

Early HGF, IL-18, IL-1RA, IP-10, and VEGF-A and post-acute MIP-1α and VEGF-A concentrations trended positively with increasing COVID-19 severity (q-values < 0.05, Jonckheere-Terpstra trend test). Increases in EGF, MIP-1β, and RANTES concentration and decreases in MIP-1α concentration over time were associated with mild rather than moderate or severe disease. Increases in MIP-1β and RANTES concentration and decreases in Eotaxin concentration over time were associated with the absence of long-term symptoms.

**Conclusions:**

Worse COVID-19 severity by 30 days was associated with higher early and post-acute period cytokine concentrations. Participants with long-term symptoms did not see resolution of cytokine responses over time.

## 1 Introduction

Severe acute respiratory syndrome coronavirus 2 (SARS-CoV-2) infections and the resulting disease, COVID-19, led to an estimated 879,100 hospitalizations and 100,800 deaths from October 2023 to September 2024 in the U.S (1). Incidence rates of hospitalization and death for individuals with the highest risk have been estimated to be 103.2 and 53.4 per 1000 person years, respectively (2). COVID-19 can lead to severe symptoms, including pneumonia and hypoxemia (3). In addition to the acute symptoms of the disease, an estimated 10.3% of individuals experience long COVID at three months following an infection (4). Long COVID symptoms can last for years and frequently include impaired cognitive function, shortness of breath, and increased fatigue (5, 6). SARS-CoV-2 is expected to remain endemic (7), yet the factors influencing disease severity and long-term symptoms are not well understood, making it important to understand the progression of the disease during and beyond the acute stage of infection.

Peripheral blood cytokines perform key roles in managing the immune response during SARS-CoV-2 infections, but also contribute to deleterious effects when their expression is dysregulated (8). Peripheral blood cytokines measured during the acute phase of infection are associated with severity of COVID-19 (9–12), but their relationships to these outcomes from later stages (i.e. more than 14 days post-infection) of infection are unclear. Additional work has examined peripheral blood cytokines and their relationship to long-term symptoms (13, 14), but again, relationships with peripheral blood cytokine concentrations during later stages of infection are underexplored. Furthermore, relationships between early and post-acute infection cytokine concentrations on long-term symptoms are poorly characterized or use shorter timeframes for longitudinal cytokine sampling. Identification of cytokines with roles in COVID-19 severity or long-term symptoms could allow for early detection of adverse outcomes, identification of intervention targets, and identify areas for further research; thus, understanding the contrast between early and post-acute cytokine concentrations on COVID-19 severity and risk of long-term symptoms remains an important topic.

U.S. Veterans disproportionately experience higher rates of severe health outcomes compared to the general population, making it important to study acute COVID-19 outcomes and long COVID in this population (15). U.S. Veterans also experience high rates of co-morbidities (15), which are associated with a greater risk of complications during a SARS-CoV-2 infection as well as long-term symptoms (16, 17). Over 130,000 U.S. Veterans tested positive for SARS-CoV-2 in the 14 months prior to October, 2024, among whom approximately 2% experienced COVID-19 associated hospitalizations (18). The Epidemiology, Immunology, and Clinical Characteristics of COVID-19 (EPIC^3^) study followed three cohorts of U.S. Veterans both with and without SARS-CoV-2 infections to understand how COVID-19 impacts Veteran’s health (19). Previous work by this group examined acute peripheral blood cytokines as predictors of COVID-19 severity in the EPIC^3^ study (20), but the contrast between early and post-acute cytokines samples has not yet been explored, nor were relationships with long-term symptoms estimated.

Here, we examined the associations between a multiplexed panel of peripheral blood cytokines and the highest COVID-19 severity within 30 days of study enrollment using data from the EPIC^3^ study. We used cytokine measurements collected during both early (days 0-7) and post-acute (days 14-42) time periods following SARS-CoV-2 infection to characterize and contrast temporal differences in these associations. We further examined associations between early and post-acute period cytokines and prolonged long-term symptoms, to estimate whether cytokine responses are modulated in an individual symptom-specific manner. Cytokine concentrations at individual time periods and longitudinal changes in cytokine concentration were used to assess relationships with cognitive impairment, fatigue, and difficulty breathing three months after study enrollment.

## 2 Materials and methods

### 2.1 Study design and population

This study utilizes the data collected by the Epidemiology, Immunology, and Clinical Characteristics of COVID-19 (EPIC^3^) study, a longitudinal, observational cohort that prospectively recruited U.S. Veterans with and without active SARS-CoV-2 infections across 16 VA medical centers from July 2020 to September 2022 (19). Three cohorts of Veterans were enrolled: inpatient participants, outpatient participants, and participants living in community living centers (CLCs). Inpatient and outpatient participants required a documented SARS-CoV-2 RT-PCR test or test order in the three weeks prior to study enrollment for inclusion in the EPIC^3^ study. Enrollment date was defined as the day that a participant provided consent to participate in the study. Questionnaires were administered at enrollment and at defined intervals following enrollment (3, 7, 14, 21, and 28 days after enrollment and 3, 6, 12, 18, and 24 months after enrollment). Peripheral blood samples were collected at the same intervals when possible.

### 2.2 Inclusion criteria

**Figure 1:**
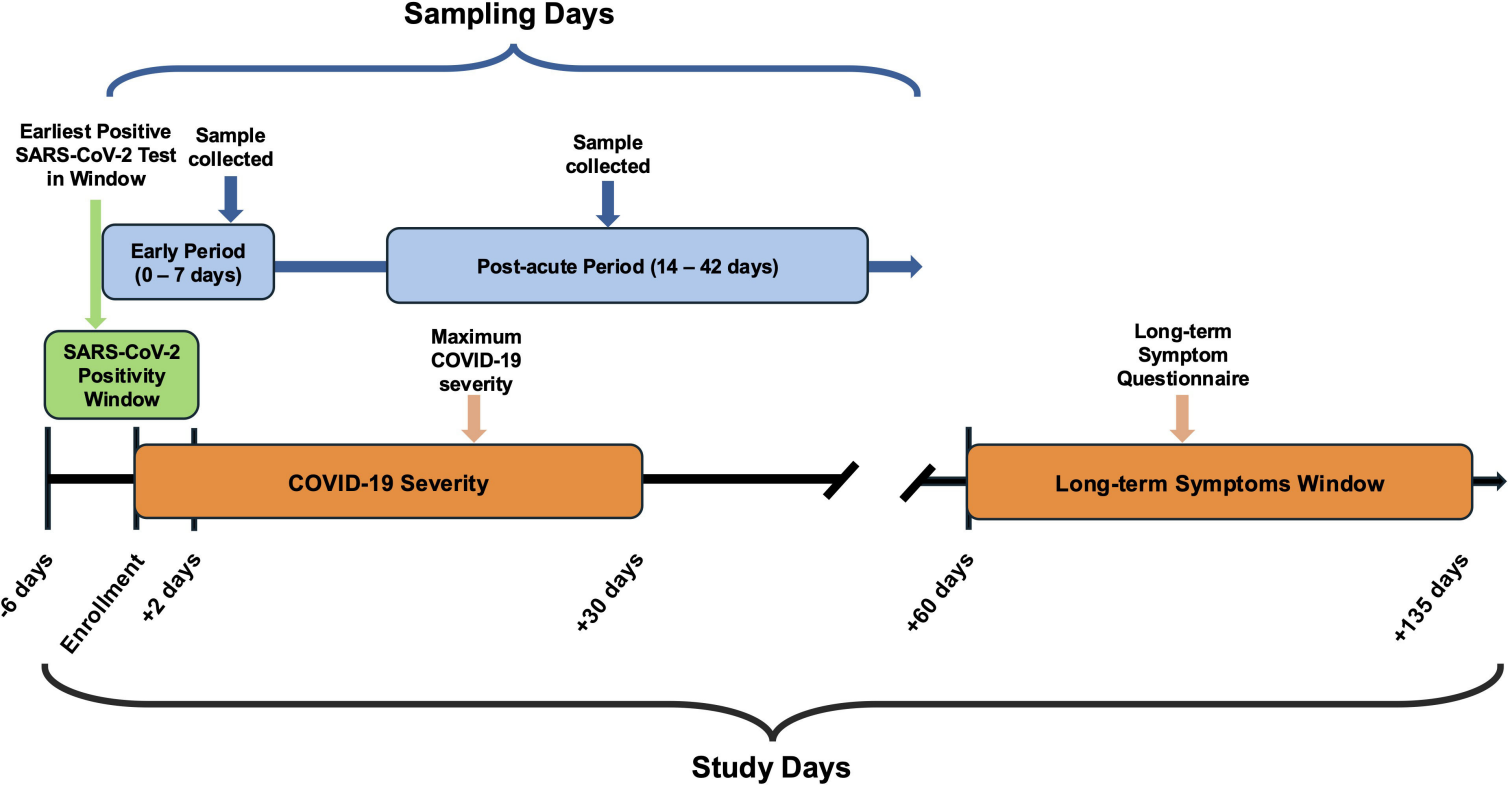
Sample collection and COVID-19 outcomes timeline. Green: SARS-CoV-2 positivity window; green arrow indicates an example earliest positive test in window. Blue: Peripheral blood sampling periods; blue arrows indicate example peripheral blood sample collection days. Orange: COVID-19 severity and long-term symptoms periods; orange arrows indicate example highest severity and long-term symptom questionnaire collection dates.

For inclusion in the current study’s analysis, participants needed to be age 18 or older at time of enrollment. To preserve sample sizes due to variability in collection of the study visit blood samples and because there was limited longitudinal data when using exclusively antigen and PCR tests to determine SARS-CoV-2 positivity, a custom SARS-CoV-2 positivity definition was used separate from the broader EPIC3 definition; participants needed to have tested positive for a SARS-CoV-2 infection via a RT-PCR, antigen, or antibody test, or had a confirmed diagnosis of COVID-19 (assigned the ICD-10 code ‘U07.1’) in the 6 days preceding the enrollment date up to the 2 days following enrollment (**Figure 1**). Furthermore, a participant must also have successful quantification of the cytokines from the peripheral blood panel in at least one of the two sampling time periods.

### 2.3 Study measures

#### 2.3.1 Exposures

Cytokines were measured from peripheral blood samples collected during early and post-acute time periods following infection. Here, the early period was defined as falling within 7 days of the first positive SARS-CoV-2 test in the positivity window (N = 387), and the post-acute period was defined as falling between days 14–42 after the first positive SARS-CoV-2 test in the positivity window (N = 191, **Figure 1**). Each participant only had one sample from a given time period. A panel of 45 cytokines were measured from peripheral blood samples: brain-derived neurotrophic factor (BDNF), Epithelial Growth Factor (EGF), Eotaxin, Fibroblast Growth Factor (FGF)-2, Granulocyte-Macrophage Colony-Stimulating Factor (GM-CSF), Growth Regulated Oncogene-alpha (GRO-α), Hepatocyte Growth Factor (HGF), Interferon (IFN)-α, IFN-γ, Interleukin (IL)-1α, IL-1β, IL-1RA, IL-2, IL-4, IL-5, IL-6, IL-7, IL-8, IL-9, IL-10, IL-12(p70), IL-13, IL-15, IL-17A, IL-18, IL-21, IL-22, IL-23, IL-27, IL-31, Interferon Gamma-Induced Protein (IP)-10, Leukemia Inhibitory Factor (LIF), Monocyte Chemoattractant Protein (MCP)-1, Macrophage Inflammatory Protein (MIP)-1α, MIP-1β, Nerve Growth Factor-beta (NGF-β), Platelet-derived Growth Factor (PDGF-BB), Placental Growth Factor (PlGF-1), RANTES (CCL5), Stem Cell Factor (SCF), Stromal Cell-Derived Factor (SDF)-1α, Tumor Necrosis Factor (TNF)-α, TNF-β, Vascular Endothelial Growth Factor (VEGF)-A, and VEGF-D. Samples were assayed on the Luminex platform at the Public Health Reference Laboratory (PHRL) within the VA Palo Alto Health Care System.

Covariates in our adjusted models included age at enrollment, sex, and Charlson Comorbidity Index (CCI) (21). Age at enrollment and sex were gathered from baseline questionnaires or electronic health record (EHR) data. CCI was calculated from EHR data in the year prior to enrollment using ICD-9 and ICD-10 diagnoses as described elsewhere (21) and excluding age from its calculation.

#### 2.3.2 Outcomes

COVID-19 severity was assessed with the Veterans Affairs Severity Index for COVID-19 (VASIC) (22) using the highest observed severity within 30 days of enrollment (**Figure 1**). The VASIC categorizes severity into 4 groups: mild, moderate, severe, and death. Disease was ‘Mild’ if a participant tested positive and had less than a 24 hour stay at the hospital or was not hospitalized; ‘Moderate’ disease included participants that had greater than a 24 hour stay at the hospital, but did not require more than low-flow oxygen therapy; ‘Severe’ disease included participants that had greater than a 24 hour hospitalization and who required high-flow oxygen therapy, intubation, mechanical ventilation, vasopressors, extracorporeal membrane oxygenation, or kidney dialysis; all-cause mortality was included in ‘Death’. We combined the severe and death categories in COVID-19 severity analyses.

Long-term symptoms of COVID-19 were assessed using questionnaire data from the month three long-term symptoms questionnaire. Due to variability in the timing of administration of the month three questionnaire, we included questionnaire results between days 60 and 135 (**Figure 1**). If a participant had multiple questionnaires during that timeframe, we used the earliest questionnaire in that timeframe. We used three primary long-term outcomes; the Patient-Reported Outcomes Measurement Information System (PROMIS) Fatigue 6a score (23, 24), PROMIS Cognitive Function 4a score (25), and the modified Medical Research Council (mMRC) Dyspnea scale (26). PROMIS scores were standardized to the general U.S. reference population and dichotomized as ‘Impaired’ if a participant’s standardized score was greater than half a standard deviation from the reference population average and ‘Unimpaired’ otherwise following recommendations from HealthMeasures (27), e.g. PROMIS fatigue was impaired if the standardized value was greater than 55. The mMRC dyspnea scale was dichotomized as ‘Impaired’ if they had a score of 1 or greater (with 1 indicating dyspnea when hurrying or walking up a slight hill) and ‘Unimpaired’ otherwise.

### 2.4 Statistical analysis

Missing and out-of-range cytokine concentrations were multiply imputed using Amelia (v.1.8.3) (28) and batch effect corrected using the ComBat function (29) in SVA (v3.56.0) (30) (supplemental methods). Distribution changes in cytokine concentration were summarized following imputation and batch effect adjustment (**Supplementary Table 1**). Cytokines with greater than 20% missingness or out-of-range concentrations were removed from the main analysis.

Correlations between each pair of cytokines were assessed by sampling period. Correlations across imputations were pooled by applying Fisher *Z* transformations to the correlation coefficients from each imputation, pooling Z scores using Rubin’s rules, and then transforming back to correlation coefficients (31–33).

We assessed trends in cytokine concentration with COVID-19 severity at each sampling period using Jonckheere-Terpstra trend tests (34, 35) as implemented in DescTools (v0.99.59) (36). As there are no well-described methods for pooling the *S* statistic across multiply imputed data, we constructed joint null distributions to estimate pooled effects (supplemental methods). Multiple comparisons were controlled using the Benjamini-Hochberg procedure. Q-values are reported in results. P-values across imputations are also summarized (**Supplementary Table 2**).

To assess whether cytokine concentration was associated with pair-wise comparisons of severity, we fit logistic regression of individual cytokine concentration on each pair of severity scores (mild vs. moderate, mild vs. severe/death, and moderate vs. severe/death), adjusting for age at enrollment, sex, and CCI. Models for each cytokine were fit separately for early and post-acute periods. Imputations were pooled using Rubin’s rules (37) as implemented in the *mice* package (v3.18.0) (38). Multiple comparisons were controlled using the Benjamini-Hochberg procedure for each severity comparison. Q-values are reported.

Separate logistic regressions were fitted for each of the cytokines and by sampling period on individual long-term symptoms comparing the odds of impairment by cytokine log_2_ concentration. These models also adjusted for age at enrollment, sex and CCI. Rubin’s rules (37) were used to pool results across imputations using *mice* package (v3.18.0) (38). Multiple comparisons were controlled for using the Benjamini-Hochberg procedure for all results of a given symptom. Q-values are reported.

We conducted Wilcoxon signed rank tests on the subset of participants that had cytokine measurements during both acute and post-acute stages of infection (N = 72). Differences in individual cytokine concentrations over time were assessed for each severity group separately. For long-term symptoms, we analyzed the change in cytokine concentration over time separately for individuals with impaired and unimpaired status for each symptom. In addition to the individual long-term symptoms, we also assessed for temporal changes in cytokine concentrations for individuals with impairment in any one of the three measures and in individuals with no impairment in any of the three long-term symptoms. To pool across imputed datasets, we constructed Monte Carlo joint null distributions of the signed rank test statistic (supplemental methods). False discovery rates were controlled with the Benjamini-Hochberg procedure. The observed p-values across imputations are also summarized (**Supplementary Tables 3**, **4**).

### 2.5 Sensitivity analysis

Cytokines that did not meet filtering criteria were included in an expanded correlation analysis using imputed and batch effect adjusted values of their concentrations.

As including multiply imputed outcomes can help reduce bias and improve power (37), we repeated all main analyses with the imputed outcomes included in the models.

Our SARS-CoV-2 positivity definition included positive antibody tests and ICD-10 code assignments and may include false positives e.g. a previous infection, diagnosis error, vaccination, etc. We conducted all main analyses in the subset of individuals that tested positive by antigen or RT-PCR test in the window around enrollment to target individuals most likely to have an active infection at enrollment. This included re-imputing and adjusting for batch effects with the altered positivity definition.

All analyses were performed using R (v4.4.1) (39).

## 3 Results

There were 503 SARS-CoV-2 positive participants with peripheral blood samples available, collected at either early or post-acute periods, for the COVID-19 severity outcome (**Table 1**). The median peripheral blood sampling time for the early period was 2 days (IQR = 0–3 days, N = 387) after a first positive SARS-CoV-2 test within the window around enrollment. The post-acute period had a median sampling time of 32 days (IQR = 29 - 35, N = 191) after the indexing SARS-CoV-2 test. There were 72 individuals that had cytokine profiling during both time periods. Most early period samples came from participants with moderate disease (51%), while post-acute period samples came from participants with mild disease (66%). The median patient age was 60 (IQR = 46 - 71) from the post-acute period compared with 67 (IQR = 59 - 73) from the early period. Participants from the post-acute period generally had lower CCI (median = 1, IQR = 0 - 3) than participants during the early period (median = 3, IQR = 1 - 5). These differences in age and CCI were reflected in the participants included in the long-term symptom subset (N = 260, **Table 2**); the median age in early period participants was 66 (IQR = 58 - 71) compared with 59 (IQR = 45 - 68) in the post-acute period, while the median CCI in early period participants was 3 (IQR = 1 - 4) compared with 1 (IQR = 0 - 3) in the post-acute period (**Table 2**). Forty-seven percent of individuals with an early sample had dyspnea at three months compared with only 37% of individuals with a post-acute sample. Twenty-four percent of participants had one long-term symptom, while only 15% and 9% of participants had two or three long-term symptoms, respectively (**Supplementary Table 5**).

**Table 1:** Participant characteristics stratified by timing of peripheral blood sample collection relative to the indexing positive SARS-CoV-2 test date.

Characteristic	Early period N = 387*^1^*	Post-acute period N = 191*^1^*	Overall N = 503*^1^*
Day Sampled	2 (0, 3)	32 (29, 35)	3 (1, 29)
Sex			
Female	32 (8.3%)	24 (13%)	48 (9.5%)
Male	355 (92%)	166 (87%)	454 (90%)
Prefer Not To Answer	0 (0%)	1 (0.5%)	1 (0.2%)
Age at Enrollment	67 (59, 73)	60 (46, 71)	65 (55, 73)
Cohort			
Community Living Center	10 (2.6%)	1 (0.5%)	11 (2.2%)
In-Patient	277 (72%)	49 (26%)	291 (58%)
Out-Patient	100 (26%)	141 (74%)	201 (40%)
VASIC Category			
Mild	78 (20%)	127 (66%)	178 (35%)
Moderate	197 (51%)	36 (19%)	211 (42%)
Severe	38 (9.8%)	9 (4.7%)	39 (7.8%)
Death	17 (4.4%)	0 (0%)	17 (3.4%)
Unknown	57 (15%)	19 (9.9%)	58 (12%)
Charlson Comorbidity Index	3 (1, 5)	1 (0, 3)	2 (1, 4)
Unknown	14	28	35 (7%)
Race			
Black or African American	158 (41%)	39 (20%)	176 (35%)
White	194 (50%)	129 (68%)	279 (55%)
Two or More Recorded Races	11 (2.8%)	10 (5.2%)	17 (3.4%)
Other	10 (2.6%)	5 (2.6%)	12 (2.4%)
Unknown	14 (3.6%)	8 (4.2%)	19 (3.8%)

*^1^*Median (Q1, Q3); n (%).

**Table 2:** Summary of participants with day 90 mMRC dyspnea, PROMIS fatigue, and PROMIS cognition quantification stratified by timing of peripheral blood sample collection.

Characteristic	Early period N = 165*^1^*	Post-acute period N = 154*^1^*	Overall N = 260*^1^*
Sex			
Female	15 (9.1%)	19 (12%)	27 (10%)
Male	150 (91%)	134 (87%)	232 (89%)
Prefer Not To Answer	0 (0%)	1 (0.6%)	1 (0.38%)
Age at Enrollment	66 (58, 71)	59 (45, 68)	63 (52, 71)
Cohort			
Community Living Center	7 (4.2%)	0 (0%)	7 (2.7%)
In-Patient	86 (52%)	33 (21%)	94 (36%)
Out-Patient	72 (44%)	121 (79%)	159 (61%)
PROMIS Cognition			
Unimpaired	129 (78%)	124 (81%)	204 (78%)
Impaired	32 (19%)	30 (19%)	52 (20%)
Unknown	4 (2.4%)	0 (0%)	4 (1.5%)
PROMIS Fatigue			
Unimpaired	120 (73%)	122 (79%)	193 (74%)
Impaired	41 (25%)	32 (21%)	63 (24%)
Unknown	4 (2.4%)	0 (0%)	4 (1.5%)
mMRC Dyspnea			
Unimpaired	73 (44%)	89 (58%)	132 (51%)
Impaired	77 (47%)	57 (37%)	107 (41%)
Unknown	15 (9.1%)	8 (5.2%)	21 (8.1%)
Charlson Comorbidity Index	3 (1, 4)	1 (0, 3)	2 (0.25, 3.75)
Unknown	12	25	30 (12%)
Race			
Black or African American	56 (34%)	30 (19%)	69 (27%)
White	93 (56%)	104 (68%)	164 (63%)
Two or More Recorded Races	6 (3.6%)	8 (5.2%)	11 (4.2%)
Other	6 (3.6%)	4 (2.6%)	7 (2.7%)
Unknown	4 (2.4%)	8 (5.2%)	9 (3.5%)

*^1^*n (%); Median (Q1, Q3).

**Figure 2:**
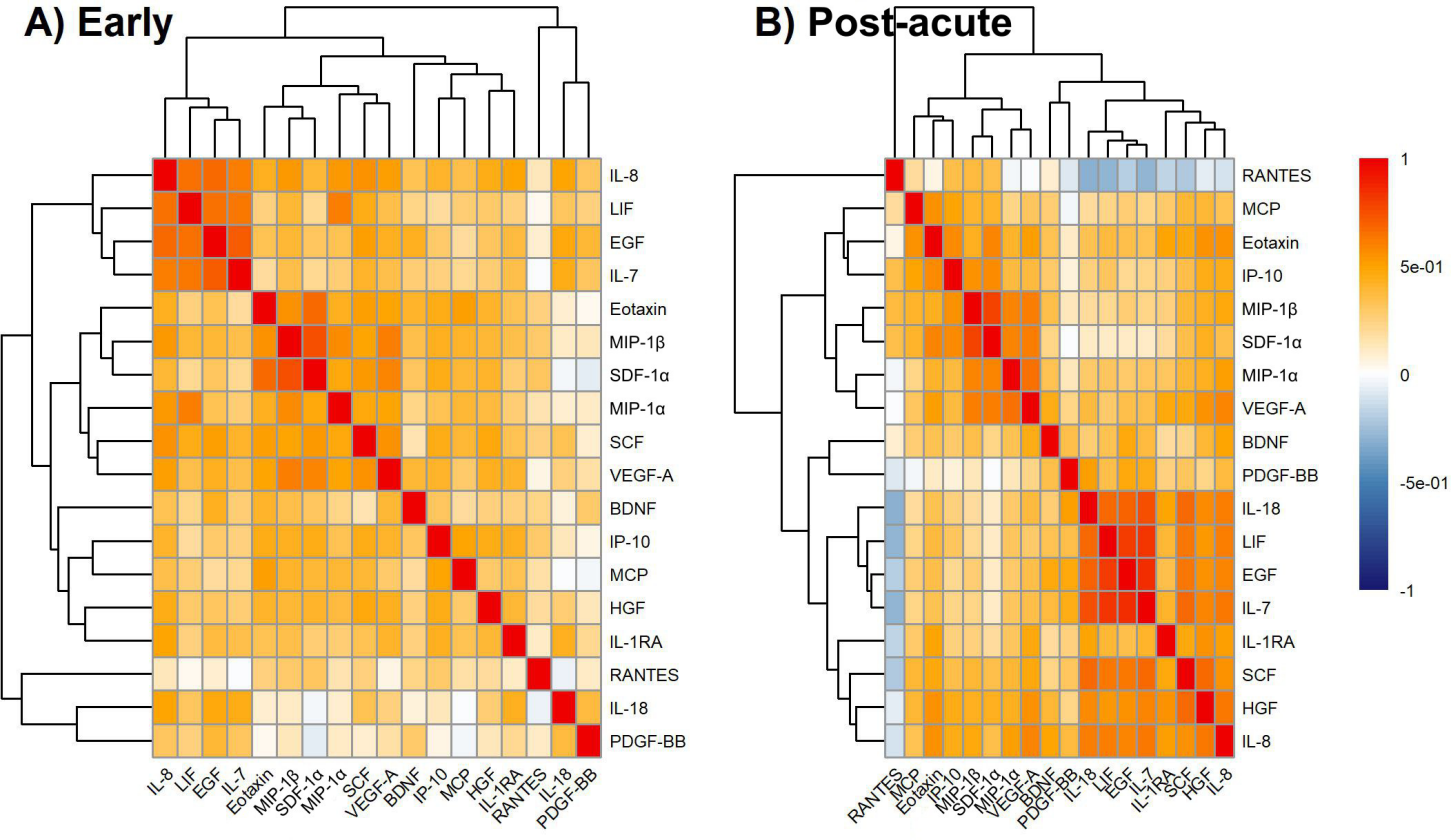
Heatmap of correlations between selected subset of cytokines in early **(A)** and post-acute **(B)** sampling periods.

There were two clusters of moderately correlated cytokines (r > 0.5 in pairwise correlations) in early period samples: LIF, EGF, IL-7, MIP-1α, IL-8, and SCF comprised one cluster and Eotaxin, SDF-1α, MIP-1β, and VEGF-A comprised the second cluster (**Figure 2A**). In the post-acute period, there were two clusters of moderately correlated cytokines present: EGF, HGF, IL-7, LIF, IL-18, SCF, and IL-8 comprised cluster one, and MIP-1β, SDF-1α, MIP-1α, and VEGF-A comprised cluster two (**Figure 2B**). RANTES was also mildly negatively correlated with most other cytokines in the post-acute period. In sensitivity analysis of all 45 cytokines profiled, there were single large clusters of moderately correlated cytokines in early and post-acute period samples, principally comprised of cytokines excluded from the main analysis, but also including EGF, HGF, IL-7, IL-8, IL-18, LIF, and SCF from the main analysis (**Supplementary Figure 1**).

**Figure 3:**
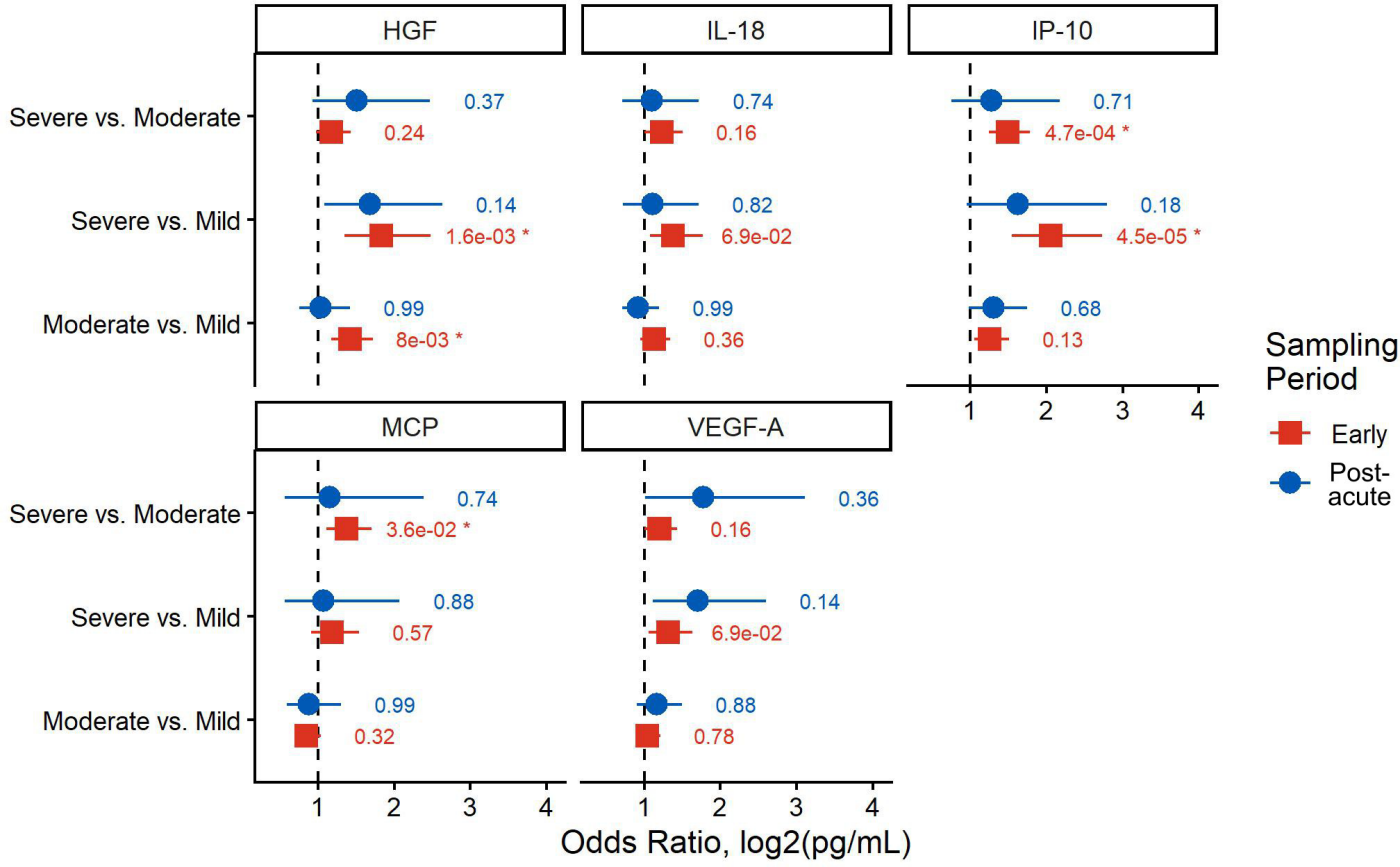
Forest plots of effect estimates from logistic regression of each log_2_ cytokine concentration (pg/mL) on VASIC severity pair-wise comparisons adjusted for age, sex, and CCI. Modelled individually for early and post-acute period samples. Only cytokines with at least one adjusted q-value below 0.1 displayed. Benjamini-Hochberg q-values displayed next to their respective estimates. Q-values below 0.05 noted with an asterisk.

**Figure 4:**
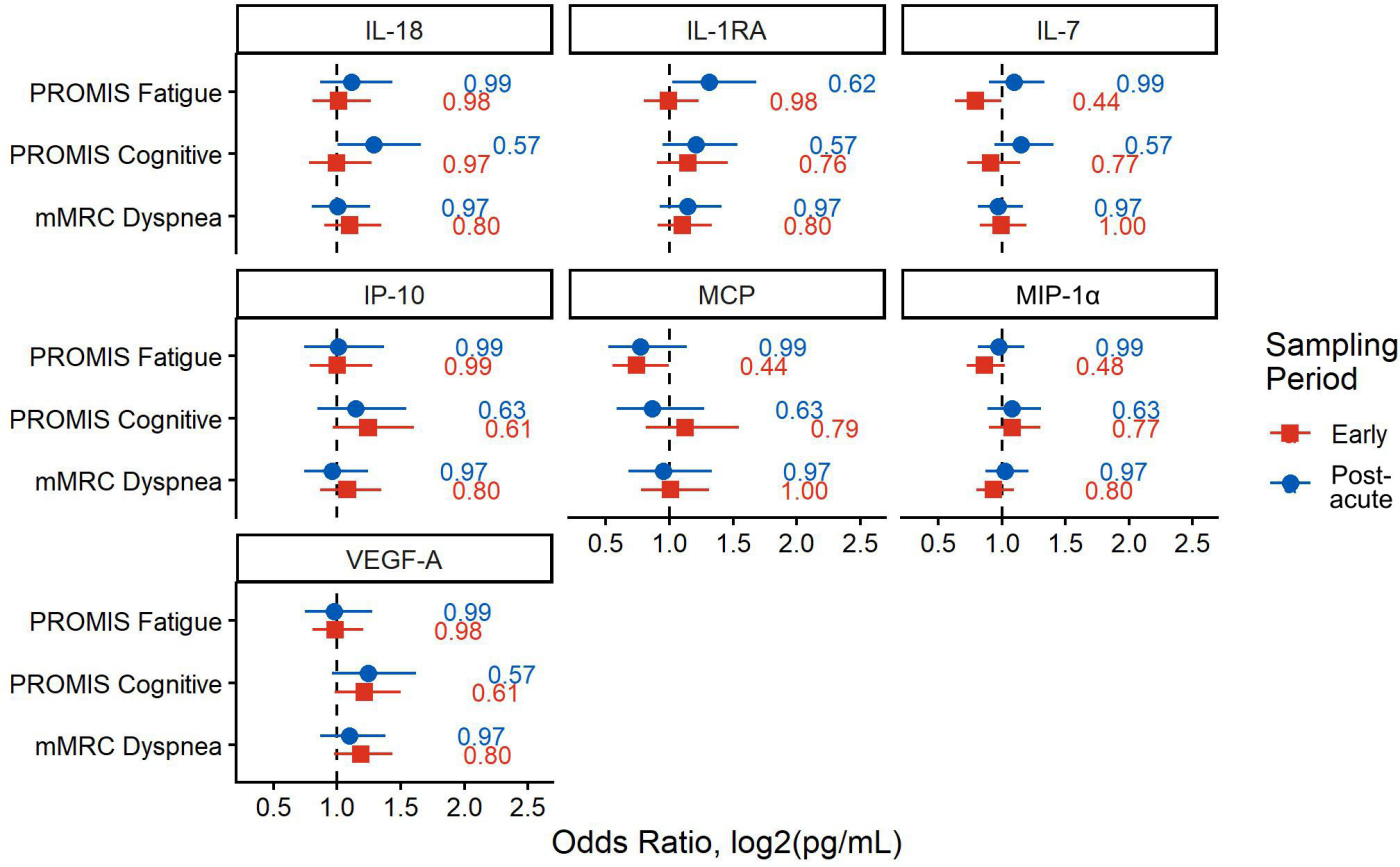
Forest plots of results of logistic regression for each log_2_ cytokine concentration (pg/mL) on day 90 long-term symptoms. Includes results from both early and post-acute infection models. Only cytokines with at least one nominal p-value below 0.1 presented. Benjamini-Hochberg q-values displayed next to their respective estimates.

Several cytokines were identified as having statistically significant trends in concentration with COVID-19 severity by Jonkheere-Terpstra tests. In post-acute samples, higher MIP-1α and VEGF-A concentrations were associated (q < 0.05) with more severe disease (**Table 3**). In early samples, higher HGF (q < 0.001), IL-1RA (q = 0.014), IL-18 (q = 0.009), IP-10 (q < 0.001), and VEGF-A (q = 0.011) concentrations were associated with more severe disease (**Table 3**). These findings were mirrored by increased odds of more severe disease with higher early period HGF, IP-10, IL-18, and VEGF-A concentrations in multiple pair-wise comparisons (**Figure 3**). Higher early MCP-1 was associated with an increased odds of more severe disease (OR 1.37, 95% CI: 1.11-1.70, q = 0.036), comparing severe to moderate disease. Multiple sensitivity analyses found similar results (**Supplementary Tables 6**, **7**; **Supplementary Figures 2**, **3**). We did not find evidence of associations between cytokine concentration and odds of long-term symptom presence in regression models in either the early or post-acute period (**Figure 4**). Sensitivity regression analysis on long-term symptoms were similarly null (**Supplementary Figures 4**, **5**).

**Table 3:** Results from the Jonckheere-Terpstra trend test; Benjamini-Hochberg q-values and direction of associated trend, e.g. an increasing association implies cytokine concentrations tended to be higher with more severe COVID-19.

	Early period		Post-acute period	
Cytokine	q-value	Direction	q-value	Direction
HGF	<0.001*	Increasing	0.53	Increasing
IL-1RA	0.014*	Increasing	0.071	Increasing
IL-8	0.386	Increasing	0.144	Increasing
IL-18	0.009*	Increasing	0.917	Increasing
IP-10	<0.001*	Increasing	0.194	Increasing
MIP-1α	0.408	Decreasing	0.002*	Increasing
MIP-1β	0.807	Increasing	0.194	Increasing
VEGF-A	0.011*	Increasing	0.049*	Increasing

Only cytokines with at least one p-value (early or post-acute period) below 0.1 displayed. Q-values below 0.05 noted with an asterisk.

We examined whether longitudinal evaluation of cytokine responses were associated with severity of disease. Longitudinal increases in EGF (q = 0.020), MIP-1β (q = 0.026), and RANTES (q = 0.020) concentration over time were associated with mild disease (**Table 4**). Decreased MIP-1α concentration over time was also associated with mild disease (q = 0.026). Sensitivity analysis using imputed COVID-19 severity additionally identified associations with decreases in Eotaxin (q = 0.009) among individuals with mild COVID-19 (**Supplementary Table 8**). No associations with changes in cytokine concentration were identified in sensitivity analysis using the strict SARS-CoV-2 positive subset (**Supplementary Table 9**), although sample sizes were smaller for both the mild (N = 10) and moderate (N = 18) disease groups than in the main analysis. Overall, these data demonstrated that changes in cytokine response were the most pronounced in individuals with mild disease.

**Table 4:** Results of Wilcoxon signed rank test by COVID-19 VASIC severity group.

Cytokine	Severity	Nominal p-value	BH adjusted q-value	Direction
BDNF	Mild	0.086	0.212	Decreased
EGF	Mild	0.002*	0.02*	Increased
Eotaxin	Mild	0.024*	0.085	Decreased
HGF	Moderate	0.059	0.353	Decreased
IL-8	Mild	0.094	0.212	Decreased
IL-8	Moderate	0.057	0.353	Increased
IP-10	Severe	0.037*	0.666	Decreased
MIP-1α	Mild	0.005*	0.026*	Decreased
MIP-1β	Mild	0.006*	0.026*	Increased
RANTES	Mild	0.001*	0.02*	Increased
RANTES	Moderate	0.005*	0.09	Increased
SCF	Mild	0.084	0.212	Increased

Direction indicates whether a cytokine had a tendency to increase or decrease from early to post-acute period. Only groups with a nominal p-value below 0.1 displayed. Mild N = 24, Moderate N = 22, Severe N = 8. P-values and q-values below 0.05 noted with an asterisk.

Next, we evaluated whether changes in cytokine responses between early and post-acute period were associated with development or protection from long COVID symptoms of fatigue, shortness of breath, or neurocognitive dysfunction three months after enrollment. We found that Eotaxin levels did not change over time in individuals with chronic fatigue or abnormal cognitive function three months after enrollment, whereas Eotaxin concentrations in individuals without these symptoms were found to decrease over time (q < 0.05, **Table 5**). Increased MIP-1β concentration over time was associated with the absence of any long-term symptoms (q = 0.029); there was no concordant increase in MIP-1β concentration over time among individuals with any symptoms (**Table 5**). Increases in RANTES concentration were associated with the absence of any long-term symptoms (q = 0.007), unimpaired cognitive function (q = 0.007), the absence of fatigue (q < 0.001), and the absence of dyspnea (q = 0.032). Sensitivity analysis using imputed long-term symptom status showed additional temporal changes in cytokine concentrations associated with long-term symptoms status, including groups with long-term symptoms (**Supplementary Table 10**). In further sensitivity analysis in the strict SARS-CoV-2 positive subset of participants, only increases in RANTES remained associated with normal cognitive function (q = 0.004), though sample sizes were appreciably smaller than in the main analysis (**Supplementary Table 11**). These data indicate that individuals with specific long-term symptoms do not see the same changes in cytokine responses as individuals without those long-term symptoms.

**Table 5:** Results of Wilcoxon signed rank test by long-term symptom group.

Cytokine	Measure	Impaired?	Nominal p-value	BH adjusted q-value	Direction
Eotaxin	Overall	Yes	0.005*	0.062	Decreased
Eotaxin	PROMIS Cognition	No	0.003*	0.049*	Decreased
Eotaxin	PROMIS Fatigue	No	0.001*	0.023*	Decreased
MIP-1β	Overall	No	0.002*	0.029*	Increased
MIP-1β	PROMIS Cognition	No	0.004*	0.05	Increased
RANTES	mMRC Dyspnea	No	<0.001*	0.032*	Increased
RANTES	Overall	No	<0.001*	0.007*	Increased
RANTES	PROMIS Cognition	No	<0.001*	0.007*	Increased
RANTES	PROMIS Fatigue	No	<0.001*	<0.001*	Increased

Direction indicates whether a cytokine had a tendency to increase or decrease from early to post-acute sampling period. Q-values presented next to respective estimates. Only groups with a q-value below 0.1 included. PROMIS Fatigue normal/impaired N = 48/10, PROMIS Cognition normal/impaired N = 48/10, mMRC Dyspnea normal/impaired N = 29/27, Overall normal/impaired N = 26/30. P-values and q-values below 0.05 noted with an asterisk.

## 4 Discussion

In this study, we explored relationships between peripheral blood cytokines and both COVID-19 severity at 30 days and presence of long-term symptoms at three months following a SARS-CoV-2 infection. In analysis of individual time periods, HGF, IL-1RA, IL-18, IP-10, and VEGF-A were associated with worsened disease severity during the early period. During the post-acute period, higher concentrations of MIP-1α and VEGF-A were also associated with more severe disease. We also saw that changes in EGF, MIP-1α, MIP-1β, and RANTES between early and post-acute periods were associated with mild COVID-19 but not with moderate or severe disease. Changes in Eotaxin, MIP-1β, and RANTES concentrations over time were associated with long-term symptom status; Eotaxin did not change over time in individuals with impaired cognitive function and increased fatigue, when their symptom-free counterparts saw decreases in Eotaxin concentration. MIP-1β and RANTES did not change over time in groups with any long COVID symptoms, yet both cytokines increased in groups with no long COVID symptoms.

In our longitudinal analysis, we observed changes in Eotaxin, MIP-1β, and RANTES responses in a long-term symptom specific manner. To our knowledge, these data are the first to demonstrate longitudinal associations between these cytokine’s responses and specific long COVID outcomes. Sbierski-Kind et al. and Ra et al. both identified associations with RANTES and long COVID at later (>1 month from infection) time points, with Sbierski-Kind et al. also finding associations with EGF, Eotaxin, MCP-1, IL-1RA, and VEGF-A (40, 41). However, both previous works used a single aggregated measure of long COVID, which may obfuscate the relationships of cytokines on specific symptoms. In other works that conducted longitudinal sampling and analysis (41–44), there were no concordant associations with the current work, except where there were null findings. In many previous works (and in our paired analysis), confounding variables were not included in models which may explain some of the conflicts in our results. However, differences in findings may be the result of distinct definitions of long COVID and specific symptoms. For example, Maciel et al. looked for multiplicative effects of count of symptoms and Gebo et al. used the presence of a single symptom as a marker of long COVID (42, 43). We identified additional longitudinal changes in BDNF, EGF, Eotaxin, HGF, and MIP-1α related to long-term symptoms in sensitivity analysis; as this moderately increased our sample sizes, particularly in groups with long-term symptoms, we were likely underpowered to detect more modest changes in cytokine concentrations. Other longitudinal works were similarly limited by small samples, suggesting that additional immune response changes related to long-term symptom occurrence have not been captured.

Associations between cytokines and long COVID or specific long-term symptoms or long COVID have been identified relative to healthy or recovered controls during acute infection and beyond, with much of this literature focusing on cytokines concentrations concurrent with long-term symptoms (14, 40, 45–52). Several cytokines have been repeatedly identified, including IL-1α, IL-6, IL-10, IP-10, and TNF-β (14, 40, 46). Among individuals that had cytokines quantified during SARS-CoV-2 acute or post-acute stages of infection, IL-1RA, IL-6, IL-8, and MCP-1 were associated with a greater risk of long-term symptoms or long COVID (42, 53). We did not detect associations in our single time period analyses of cytokine responses, to the extent that a given cytokine was included in our analysis following filtering. Interestingly, Lu et al. also did not find any associations in risk of long COVID at 4 months post-infection in any of the quantified cytokines sampled during acute and post-acute infection among SARS-CoV-2 positive individuals not sampled based on initial hospitalization (54). These data suggest that measurement of single cytokine responses may not be useful for predicting long COVID symptoms.

Our findings may provide insight into the biology of long-COVID. There has been some work demonstrating a neuro-protective effect of RANTES following ischemic stroke (55, 56), so increases in RANTES among individuals without long-term symptoms in the present study potentially reflects a protective effect of the chemokine against cognitive impairment. RANTES has also been shown to reduce viral load in chronic infections (57), and stimulate expression of growth factors (55), providing a potential means to aid in the repair of pulmonary damage following infection. Decreased Eotaxin indicates a transition from the innate to adaptive immune response following infection, and it is also associated with cardiovascular disease and neurodegeneration (58); this may suggest sustained elevation in Eotaxin contributes to the presence of long-term symptoms like cognitive impairment through similar mechanisms. MIP-1β is strongly induced by IFN-γ, which is a critical aspect of the SARS-CoV-2 response. It may be that lack of increases in MIP-1β indicate a relatively poor adaptive immune response, which is critical for resolution of SARS-CoV-2-induced inflammation. Immune characteristics such as immune cell phenotypes, hyperactivation of immune cells, or metabolomic profiling may improve the prognostic value of cytokine quantification on COVID-19 severity and future long-term symptoms. Our findings of symptom-specific cytokine responses leaves the open question of whether variation in these other immune characteristics lead to the development of specific long-term symptoms. Other works have begun to examine the roles of these other factors, especially with respect to COVID-19 severity (51, 59). Examining the interplay between our findings and these factors will be important for developing our biological understanding of long COVID.

Our individual time period findings give novel insight into trends in cytokine response during post-acute infection and support other early period findings. Several studies have examined early/acute peripheral blood cytokine concentrations on COVID-19 severity, with IFN-α, IL-1β, IL-6, IL-8, IL-10, IL-18, IP-10, and TNF-α in particular being routinely identified with risk of greater disease severity or mortality (9, 11, 12, 20, 60–65). We saw evidence of early period HGF, IL-1RA, IL-18, IP-10, MCP-1, and VEGF-A being associated with an individual’s risk of more severe disease, as well as IL-8 in sensitivity analysis, which is supported by similar findings (9, 12, 63, 65–68). Associations between post-acute stage cytokine responses (i.e. greater than 2 weeks post-infection) and COVID-19 severity are underexplored (61, 63, 67). We found that higher post-acute MIP-1α and VEGF-A levels were associated with more severe disease, suggesting that sustained elevation of these cytokines beyond early infection is related to severe disease; elevated post-acute MIP-1α likely indicates that there is ongoing inflammation in more severe disease and perhaps a non-specific response to infection (69), while high VEGF-A in severe disease may result from COVID-associated hypoxia and tissue damage (70). Ling et al. and Young et al. showed that these cytokines are associated with disease severity at more intermediate time periods (days 8-21) following infection (9, 63); our post-acute period findings are suggestive that these cytokines remain dysregulated longer than previously understood.

We found evidence that tracking cytokine concentrations over time was associated with acute disease outcomes. Ling et al. found evidence of changes in cytokine concentration from early (days 0-7) and late (days 8-12) infection in IL-1RA and IP-10 in moderate and severe disease, respectively (9); whereas, in the present study neither was associated with changes from early to post-acute time periods. Notably, the ‘late’ sampling window in Ling et al. is not included in (and was prior to) the present study’s post-acute period and decreases in IP-10 were nominally associated with severe disease in the present study. In other studies, IL-18 decreased among individuals with moderate COVID-19 while they remained elevated over the first three weeks post infection in those with severe disease (67). We did not see evidence of any changes in IL-18 in any severity group in this study, nor did we detect any trend in IL-18 levels across severity groups during post-acute infection. Both Lucas et al. and the present study identified longitudinal changes in RANTES among more mild/moderate COVID-19, although Lucas et al. found that it decreased over time, whereas, we found that it increased over time (67). We further identified changes in EGF, MIP-1α, and MIP-1β in our longitudinal analysis, none of which were identified in other longitudinal analyses (9, 10, 17, 60, 65), although these results are supported by other cross-sectional findings (9, 63). A longitudinal reduction in MIP-1α concomitant with increased MIP-1β in mild COVID-19 disease may suggest a controlled transition from acute inflammation into adaptive recruitment of lymphocytes infection that is not present in more severe disease, due to MIP-1α’s preferential recruitment of CD8+ T cells and MIP-1β’s recruitment of activated CD4+ T cells (69). Increases in EGF among mild disease is also suggestive of a regulated transition from acute disease to convalesce as the body begins to repair damaged tissue (71), though there is some evidence that over-expression of EGF receptors may contribute to pulmonary fibrosis following SARS-CoV infection (72). The absence of these changes in cytokine concentration for the moderate and severe groups suggests that impaired normalization of cytokine responses after infection may predispose individuals for more severe disease. Though notably, nominal associations with longitudinal changes in Eotaxin, HGF, IP-10, and RANTES were seen in groups with moderate or severe COVID-19 in sensitivity analysis, so our longitudinal data may also have been underpowered to detect changes in more severe disease.

While many previous studies were only able to examine severity in hospitalized individuals, particularly at the start of the SARS-CoV-2 pandemic, the current study recruited participants from all patients seeking care at a VHA facility allowing us to observe individuals with asymptomatic, mild, and severe disease. Age, sex, and co-morbidities are strongly associated with both COVID-19 severity and development of long COVID and affect basal plasma cytokine concentrations (17, 73, 74); we were able to adjust for these factors in a subset of our analyses in order to reduce bias in our effect estimates. Our long-term symptom analyses examined specific symptoms individually which helps to estimate specific associations between cytokine responses and long COVID symptoms that may be obscured by aggregating across all symptoms. This study also benefits from a relatively large sample size in the analyses from individual sampling periods. Furthermore, there has been limited work done to characterize any unique immune response in U.S. Veterans infected with SARS-CoV-2.

This study has several key limitations. The participants included in the early and post-acute sampling periods had incomplete overlap, and differences in associations between early and post-acute cytokines may not be solely attributable to progression of the infection, as participants that contributed peripheral blood samples at the post-acute period tended to be younger and were mainly recruited as out-patients. Furthermore, SARS-CoV-2 infection status was assessed up to three weeks before enrollment in the broader EPIC^3^ study and the present study’s positivity definition did not exclude individuals that had earlier positive SARS-CoV-2 tests, so sampling time period reflects study sampling windows rather than infection stages. We addressed this by conducting sensitivity analysis using a strict positivity definition that represents active infections in the days around enrollment; the results of this analysis are broadly concordant with the findings of the main analysis where sample sizes were sufficiently large. Due to an abundance of out-of-range concentrations in specific cytokines and known batch effects, we were unable to include the full set of 45 cytokines quantified in the final analysis, including some cytokines that have strong support for associations with COVID-19 severity (e.g. IL-6, IL-1β, and TNF-α). The exclusion of these cytokines diminishes our ability to assess the immune response in ensemble, as interactions between these factors are likely to influence the presentation of disease (e.g. antagonistic action of IL-1RA to IL-1α/β). However, we found that many of the excluded cytokines were correlated with others associated with severity and long-term symptoms, suggesting that our results have similarity with other published results (9, 12, 14, 40, 46, 51, 60–65). Additionally, exploration of cytokine associations with long-term symptoms was limited to symptoms around three months post-infection and may not hold over longer timeframes.

There are several areas for future research that would complement the findings of this study. Factors such as SARS-CoV-2 variant and SARS-CoV-2 vaccination status are associated with COVID-19 severity (75, 76), and these factors may be associated with the presence of long-term symptoms as well. Other immune factors (overactive immune cells, antibodies, etc.) or biological responses (protease activity, metabolites, proteome) may contribute to the development of severe COVID-19 and/or long-term symptoms or provide pathways for how cytokine responses affect these outcomes; future studies would benefit from a deeper exploration of these factors and the interplay between them and cytokine responses found in the present study. Since the sampling periods in the present study represented relatively broad periods that may not reflect the specific temporal shifts in cytokine levels, future work would also benefit from examining more granular sampling stages particularly during early infection. Additionally, associations between long-term symptoms and peripheral blood cytokines may change depending on time of long-term symptom determination, and thus examining longer timeframes may be useful in understanding the etiology of long COVID. Finally, research contrasting the immune responses of non-Veterans to that of the Veteran population could provide insight into additional disparities COVID-19 outcomes between these groups.

Our study examined the relationships between peripheral blood cytokines during early and post-acute periods following a SARS-CoV-2 infection on risk of COVID-19 severity and development of long-term symptoms among the EPIC^3^ cohort. We find evidence of specific cytokines associated with both COVID-19 severity and the presence of long-term symptoms at 3 months as disease progressed from early to post-acute periods following infection. These results support previous findings and may provide additional prognostic value for identifying individuals at the highest risk of adverse outcomes.

## Data Availability

The data underlying this study are available upon request from the U.S. Department of Veterans Affairs from qualified VA and non-VA investigators working for nonprofit, academic, and research centers under controlled access procedures via the study repository’s application process. Access is subject to controlled-access procedures through the study repository’s application process, including applicable VA, IRB, privacy, security, and data-use approvals. Inquiries can be directed to the corresponding author.
