# Supplementary material for: Peripheral blood cytokines during early and post-acute stages of SARS-CoV-2 infection are associated with disease severity and long-term symptoms
Source: Front Immunol. 2026 Jul 15;17:1870109. doi: 10.3389/fimmu.2026.1870109 (PMC13415590; doi:10.3389/fimmu.2026.1870109)
Supplement: Supplementary file 1 [file Supplementaryfile1.docx]

The authors of this manuscript would like to thank and acknowledge the following individuals on the CSP #2028/EPIC^3^ Study for their contribution towards this manuscript:

Study Chairs

**Jennifer S. Lee, MD, PhD, CSP #2028 Co-Chair**

**Jennifer M. Ross, MD, MPH, CSP #2028 Co-Chair**

**Javeed A. Shah, MD, CSP #2028 Co-Chair**

Study Co-Investigators

**Mihaela Aslan, PhD, CSP #2028 Co-Investigator**

**Kelly Cho, PhD, MPH, CSP #2028 Co-Investigator**

**J. Michael Gaziano, MD, MPH, CSP #2028 Co-Investigator**

**Mark Holodniy, MD, CSP #2028 Co-Investigator**

**Christine M. Hunt, MD, MPH, CSP #2028 Co-Investigator**

**Anna M. Korpak, PhD, CSP #2028 Co-Investigator**

**Dawn T. Provenzale, MD, MS, CSP #2028 Co-Investigator (former)**

**Christina Williams, PhD, MPH, CSP #2028 Co-Investigator**

Baltimore

*Scientific*

**Mary-Claire Roghmann, MD, MS, Local Site Investigator**

**Karen (KC) Coffey, MD, MPH, Co-Local Site Investigator**

**Leslie (Les) Katzel, MD, PhD, Co-Local Site Investigator**

*Operations*

Michelle Newman, BSN, Research Coordinator

Gwen L. Robinson, MPH, Research Coordinator

Boston

*Scientific*

**Eric Garshick, MD, MOH, Local Site Investigator**

**Emily Wan, MD, MPH, Co-Local Site Investigator**

*Operations*

Emma Busenkell, BS, Research Coordinator (former)

Selena Chom, MPH, Research Coordinator (former)

Christina Collins, MPH, Research Coordinator (former)

Colleen Hynes, RN, Research Nurse (former)

Demerise Johnston, MPH, Research Coordinator

Erin McHugh, BS, Research Assistant (former)

Peter Rivoira, BA, NODES Operations Manager

Olivia Sterns, BS, Research Assistant (former)

John (Jack) Sweeney, BS, Research Assistant (former)

Caroline Truland, RN, BSN, BSBA, NODES Research Nurse

Makaila Wall, BS, NODES Associate Director of Operations

Pantel Vokonas, MD, Medical Monitor

Cathy Zhang, BS, Research Assistant (former)

Cleveland

*Scientific*

**Federico Perez, MD, MS, Local Site Investigator**

**Robin L.P. Jump, MD, PhD, Co-Local Site Investigator**

**Robert Bonomo, MD, Co-Investigator**

**David Canaday, MD, Co-Investigator**

**Margaret Tiktin, RN, NP, DNP, Co-Investigator**

*Operations*

Sara Abdelrahim, MBBS, Research Coordinator (former)

Taissa A. Bej, MS, Research Coordinator

Janet Briggs, RN, BSN, MSN, Research Coordinator (former)

Elizabeth Delancey-Niksa, RN, BSN, Research Nurse (former)

Oteshia Hicks, BA, Research Coordinator

Corinne Kowal, BS, Research Coordinator

Alexandria (Alex) Nguyen, MS, Research Coordinator

Lisa Padro, BSN, PMH-BC, Research Coordinator

Dallas

*Scientific*

**Roger Bedimo, MD, MS, Local Site Investigator**

**Rohit Manaktala, MD, Co-Local Site Investigator**

*Operations*

Erik Guajardo, BA, CCRP, NODES Quality Assurance Manager

Antoinette Hamilton, BS, Research Coordinator (former)

Lisa Jones, MS, NODES Quality Assurance Manager (former)

Marcia Keller-Ray, Research Coordinator

Angela Dela Llana, BSN, RN, Research Coordinator (former)

Jacob Mathew, Research Coordinator (former)

Jennifer (Jen) McClure, BSN, RN, NODES Associate Director of Operations

Erick Meermans, BS, Research Coordinator (former)

Erin Messick, MS, Research Coordinator (former)

Dindi Moore-Matthews, MS, Research Coordinator (former)

Van Nguyen, BS, Research Coordinator (former)

Abeer Zein, BS, Research Coordinator (former)

Denver

*Scientific*

**Lindsay Nicholson, MD, Local Site Investigator**

**Mary Bessesen, MD, Co-Local Site Investigator**

*Operations*

Rosa Cunningham, LPN, BS, MHA, Research Coordinator (former)

Teresa Derian, RN, Research Coordinator (former)

Theresa Dunn, MS, Research Coordinator (former)

Camila Hanson, BS, Research Coordinator (former)

Kelsey Moore, RN, BSN, Research Coordinator (former)

Kimberly Owens, MPH, CCRC, NODES Associate Director of Operations

Cameron Rogowski, BS, Research Coordinator (former)

Janel Vigil, RN, BSN, Research Coordinator (former)

Anna Wyrwa, RN, BSN, MSN, Research Coordinator

Durham

*Scientific*

**Micah McClain, MD, PhD, Local Site Investigator**

**Ephraim Tsalik, MD, PhD, Local Site Investigator (former)**

**Christopher Woods, MD, MPH, Co-Local Site Investigator**

**James Everhart, DO, Co-Investigator** **(former)**

**Christopher Hostler, MD, MPH, Co-Investigator**

**Maria Joyce, MD, PhD, Co-Investigator**

*Operations*

Jack Anderson, BS, Research Assistant (former)

Marline (Marlena) Brown, BS, Research Technician

Lynette Gehlhausen, RN, BSN, Research Nurse (former)

Amanda Hittinger, BSN, RN, Research Nurse (former)

Sara Hoffman, RN, BSN, Research Nurse (former)

Tyffany (Evans) Locklear, BS, BA, Research Coordinator (former)

Maria Miggs, BS, Research Coordinator (former)

Deborah Murray, BS, Research Coordinator (former)

Bradly (Brad) Nicholson, PhD, Lab Manager

Ashlyn Press, MPH, Program Manager (former)

Jaspreet Reen, MPH, Program Manager (former)

Delisa Robinson, BS, Research Coordinator (former)

Gainesville

*Scientific*

**Gary Wang, MD, PhD, Local Site Investigator**

**Amy Vittor, MD, PhD, Co-Local Site Investigator**

**Asmita Gupte, MD, Co-Investigator**

**Alaina Ritter, MD, Co-Investigator**

*Operations*

Leslie Brown, BA, Research Coordinator (former)

Tempa Curry, RN, Research Coordinator

Laura Dixon, BSN, Research Assistant (former)

Jennifer Gollwitzer, MSN, Research Coordinator

Rebecca Kokot, Research Assistant (former)

Debra Robertson, RN, Research Coordinator (former)

Taylor Simon, BS, Research Assistant (former)

Juliana Venetucci, MS, Research Assistant (former)

Elizabeth Vo, Research Assistant (former)

Little Rock

*Scientific*

**John Theus, MD, Local Site Investigator**

**Ryan Dare, MD, Co-Investigator**

*Operations*

Jesse Byrd, BA, Research Coordinator (former)

Adam Lallier, CRC, Research Coordinator (former)

Kristin Miller, BSN, Research Coordinator (former)

Betty Ussery, CCRC, Research Coordinator

Milwaukee

*Scientific*

**Sheran Mahatme, DO, MPH, Local Site Investigator**

**Nathan Gundacker, MD, Co-Local Site Investigator**

**Javeria Haque, MD, Co-Local Site Investigator**

*Operations*

Kasey Kallio, MSN, RN, Research Coordinator

Julie Rieder, CMA (AAMA), CCRC, NODES Associate Director of Operations (former)

Colleen Veenendaal, RN, Research Coordinator

Aprille Walker, BA, Research Coordinator

Palo Alto

*Scientific*

**Harman Paintal, MBBS, Local Site Investigator**

**Elizabeth (Lisa) Le, MD, Co-Local Site Investigator**

**Matthew (Matt) Stevenson, MD, Co-Local Site Investigator**

*Operations*

Sadaf Ahmed, MPH, Research Coordinator (former)

Karen Bratcher, MSN, RN, NODES Associate Director of Operations (former)

Ashley Langston, MS, MA, CRC, Research Coordinator (former)

Olga Livingston, Research Coordinator (former)

Edgardo A. Gamarra Monteverde, MBA, MPH, NODES Associate Director of Operations

Elena Nikolaev, NODES Quality Assurance Manager (former)

James Quinn, Research Coordinator (former)

Ann Roseman, BA, Research Coordinator

Philadelphia

*Scientific*

**Stuart Isaacs, MD, Local Site Investigator**

**Joshua (Josh) Baker, MD, MSCE, Co-Local Site Investigator**

**Kyong-Mi Chang, MD, Co-Local Site Investigator**

**Jeffrey Doyon, MD, PhD, Co-Investigator** **(former)**

**Katherine Gardner, MD, Co-Investigator** **(former)**

**Mary Hofmann, MD, RN, Co-Investigator**

**Darshana Jhala, MD, Co-Investigator**

**David Stern, MD, Co-Investigator** **(former)**

**Laura Su, MD, PhD, Co-Investigator**

*Operations*

David Azizi, BA, Research Coordinator (former)

Juliana Bonilla, BA, Research Coordinator (former)

Caleigh Doherty, BS, Research Coordinator (former)

Rachel Gillcrist, BA, Research Coordinator (former)

Criswell Lavery, MA, Research Coordinator (former)

Will Leach, MA, Research Coordinator (former)

Lynne Mancini, RN, MSN, BSN, Research Coordinator (former)

Lizbeth Novelo, BA, Research Coordinator (former)

Mariana Olave, BA, Research Coordinator

Mary Valiga, RN, Research Coordinator (former)

Sarah Wetzel, MPH, BS, Research Coordinator

Muhammad Zahid, MD, Research Coordinator (former)

Portland

*Scientific*

**Christopher (Chris) Pfeiffer, MD, MHS, Local Site Investigator**

**Marissa Maier, MD, Co-Investigator**

**Angela (Holly) Villamagna, MD, Sub-Investigator** **(former)**

*Operations*

Antwan Baker, MS, Research Coordinator (former)

Alexandra (Pitts) Bennett, BS, Research Coordinator (former)

Hannah Flegal, BA, Research Assistant (former)

Jennifer Green, BA, Research Coordinator (former)

Tawni Kenworthy-Heinige, BS, NODES Associate Director of Operations (former)

Erik Mauk, BS, Research Assistant

Laura Onstad, RN, BS, Research Coordinator (former)

Kevin Osborn, BS, BA, Research Coordinator

Ginger Sullivan, AS, CMA, Research Assistant

Michael Tanaka, BA, Research Coordinator

Deanna Ternes, BS, Research Coordinator (former)

Senta Wiederholt, BA, Research Assistant (former)

Lorrinda Zahl, AA, CPT, Research Assistant

Salt Lake City

*Scientific*

**Patrick (Pat) Powers, MD, Local Site Investigator**

**Julia Lewis, DO, Co-Local Site Investigator**

**Emily Beck, MD, Co-Investigator** **(former)**

**Sean Callahan, MD, Co-Investigator (former)**

**Laura Certain, MD, PhD, Co-Investigator** **(former)**

**Barbara Jones, MD, Co-Investigator (former)**

**Mustafa Mir Kasimov, MD, Co-Investigator** **(former)**

**Lynn Keenan, MD, Co-Investigator**

**Robert Paine III, MD, Co-Investigator (former)**

**Gregory Radin, MD, Co-Investigator (former)**

**Karl Sanders, MD, Co-Investigator (former)**

*Operations*

Jean Brooks, MSN, RN, CCRC, ACRP-PM, NODES Nurse Manager (former)

Brenda Hernandez, MBA, BA, Research Coordinator

Craig High, MS, Research Coordinator (former)

Vinay Kumaran, MBBS, MPH, CCRC, Research Coordinator (former)

Adam Nehls, BS, Research Coordinator (former)

Christina Nessler, MS, CCRC, NODES Operations Manager

Haleisha Power, BS, Research Coordinator (former)

Jason Ray, BBA, Research Assistant (former)

Valentino Rodriguez, BS, Research Coordinator (former)

Kaylene Russell, MPH, Research Assistant (former)

Kandi Velarde, MPH, CCRC, NODES Associate Director of Operations

San Antonio

*Scientific*

**Patrick Danaher, MD, Local Site Investigator**

**Antonio Anzueto, MD, Co-Local Site Investigator**

*Operations*

Joanne Holloway, RN, CCRC, Research Coordinator

Michele Paprocki, RN, Research Coordinator (former)

Seattle (site)

*Scientific*

**Kristina Crothers, MD, Local Site Investigator**

**McKenna Eastment, MD, MPH, Co-Local Site Investigator**

**Javeed Shah, MD, Co-Local Site Investigator**

**Arti Tayade, MD, MBBS, Co-Investigator**

**Luis Tulloch-Palomino, MD, Co-Investigator**

*Operations*

SueAnn Brickle, Research Coordinator

Joseph (Joe) Gylys-Colwell, BS, Research Coordinator (former)

Neelab (Amina) Kamiab, BS, BA, Research Assistant

John Kundzins, BS, Research Coordinator

Troy Layouni, MPH, Research Coordinator

Jacob Martin, BA, Research Coordinator (former)

Hasanah McCauley, BS, Research Coordinator (former)

Cassandra (Cassie) Stubbe, MSc, NODES Quality Assurance Manager

Rachel Tesoro, BS, Research Assistant

Pandora Lucrezia (Luke) Wander, MD, MS, FACP, Staff Physician

Kristin Wojtowicz, BS, Research Coordinator

West Haven

*Scientific*

**Shaili Gupta, MBBS, Local Site Investigator**

**Richard Sutton, MD, PhD, Co-Local Site Investigator**

*Operations*

David Ardito, Research Coordinator

Jessica O’Donovan, BA, Research Coordinator

Patricia Pelham, RN, Research Nurse

Danielle Plank, Research Coordinator

Alicia Roy, BA, Research Coordinator

Gary Stack, MD, Lab Manager

Christine Summers, MA, Research Coordinator

Seattle Coordinating Center

*Scientific*

**Nicholas L. Smith, PhD, Coordinating Center Director**

**Jonathan Sugimoto, PhD, Project Director (former)**

**Anna M. Korpak, PhD, Lead Biostatistician**

**Aaron Baraff, PhD, Biostatistician**

*Operations*

Jonathan Adams, PhD, National Study Coordinator (former)

Morgan Bergerud, BS, Research Assistant (former)

Christopher (Chris) Bromberg, MA, Research Coordinator (former)

Alexandra Fox, MSIS, Data Analyst

Helen Haile, BS, Research Specialist (former)

Tess Harpur, MPH, Research Coordinator (former)

Liuye Huang, MHS, Research Specialist

Heidi Hummel, PhD, Project Manager (former)

Samin Kamal, MS, Research Specialist (former)

Gabrielle LaBazzo, MPH, Research Specialist (former)

Xumin Li, MS, Research Specialist (former)

Cindy Liu, BA, Program Manager

Calen Mendall, MS, Research Specialist (former)

Jordanna Midthun, MPH, Research Coordinator

Kathryn Moore, PhD, Data Manager (former)

Daniel (Dan) Morelli, BA, Program Manager

Kytlan Morgan, BA, Research Assistant (former)

Geun-woo Oh, BA, Research Assistant (former)

Vivek Pakanati, MPH, Research Coordinator

Do Yeon (Doyeon) Park, MS, MPH, Research Specialist (former)

Rachel Sanders, BS, BA, Research Specialist (former)

Katie Schroeder, BS, Research Specialist

Nicholas (Nick) Simeti, MPH, Research Coordinator

Chad Sisemore, MS, Data Analyst (former)

Jennifer (Jen) Sporleder, BS, Associate Center Director, Research Operations

Adrienne Tanus, MPH, Project Manager, Research Operations (former)

Sarah Thiel, Research Assistant (former)

Tija Tippett, BS, Research Assistant (former)

Tracy Wang, MAS, Data Analyst

Gabriela Webb, BS, Research Assistant (former)

Katrina Wicks, MPH, Data Manager (former)

Deanna Wilson, MPS, National Study Coordinator (former)

Sarah Yarborough, MPH, Research Specialist (former)

Haley Dixon, MPH, Research Coordinator

Summer Gardner, MSIM, Data Analyst

Alexandra Schmidt, MPH, Data Analyst

Sahra Mohazzab-Hosseinian, PhD, Epidemiologist

Mario Trejo, PhD, Epidemiologist

Executive Committee

**Michael Boeckh, MD, PhD, CSP #2028 Executive Committee Member**

**Kyong-Mi Chang, MD, CSP #2028 Executive Committee Member**

**Elizabeth (Lisa) Le, MD, CSP #2028 Executive Committee Member**

**Yoselin Ordonez Suarez, PharmD, CSP #2028 Executive Committee Member**

**Julie Parsonnet, MD, CSP #2028 Executive Committee Member**

**Jonathan Sugimoto, PhD, CSP #2028 Executive Committee Member**

**Christopher (Chris) W. Woods, MD, MPH, CSP #2028 Executive Committee Member**
