# Supplementary material for: Peripheral blood cytokines during early and post-acute stages of SARS-CoV-2 infection are associated with disease severity and long-term symptoms
Source: Front Immunol. 2026 Jul 15;17:1870109. doi: 10.3389/fimmu.2026.1870109 (PMC13415590; doi:10.3389/fimmu.2026.1870109)
Supplement: Supplementary file 2 [file Supplementaryfile2.docx]

**Supplemental Methods**

Due to known sample clustering traced back to a hardware malfunction and subsequent repairs of the Luminex platform, we performed batch effect correction based on whether a sample was processed before or after the malfunction and repair. To improve imputation and batch effect correction we conducted these steps on all study samples (N = 1284) assessed on the Luminex platform. As our selected batch correction method required complete data, we imputed all missing or out-of-range cytokine (log_2_ transformed) concentrations, VASIC severity values, and long-term symptoms values. We used the expectation-maximization algorithm implemented in Amelia (v1.8.3) [28] to impute 10 datasets. Then, to adjust for batch effects we used the ComBat function [29] as implemented in the SVA package (v3.56.0) [30]. We included sampling time period, CCI, SARS-CoV-2 positivity, VASIC severity, and the three long-term symptoms in the model to preserve biological variation that might be affected by batch effect adjustment. Cytokines were filtered out from the analysis if they had greater than 20% overall out-of-range or missing values pre-imputation. After filtering, 18 cytokines remained in the main analysis. Missingness was added back to severity and long-term symptom outcomes following imputation in the main analysis.

To pool results across multiply imputed data, we constructed Monte Carlo joint null distributions of the Jonckheere-Terpstra test statistic and compared this to the average test statistic across imputations. The joint null distribution of test statistics was constructed by randomly shuffling severity labels and applying the same permutation to each imputation; the average of these test statistics was recorded, and the permutation process was repeated over 20,000 iterations. The observed average test statistic was compared to this null distribution to generate a two-sided p-value.

To pool results across imputed datasets in Wilcoxon signed rank tests, we again constructed Monte Carlo joint null distributions of the signed rank test statistic with the same method as with the Jonckheere-Terpstra tests. The joint null distribution of test statistics was constructed by randomly generating signs and applying these to the differences in cytokine concentration between early and post-acute sampling periods. The same allocation of signs was applied to each imputed dataset, and the average of these test statistics was recorded. The random sign allocation process was repeated over 20,000 iterations to generate a final joint null distribution. The observed average test statistic was compared to this distribution to generate a two-sided p-value.

**Supplemental Tables and Figures**

Supplemental Table 1. Distribution of cytokine concentrations in early (a) and post-acute (b) peripheral blood sampling time periods. Baseline log_2_ concentrations and mean of imputed, batch effect corrected, log_2_ concentrations of cytokines summarized by their median and interquartile range. Note, that this pooling ignores the between-imputation variance in concentration estimates.


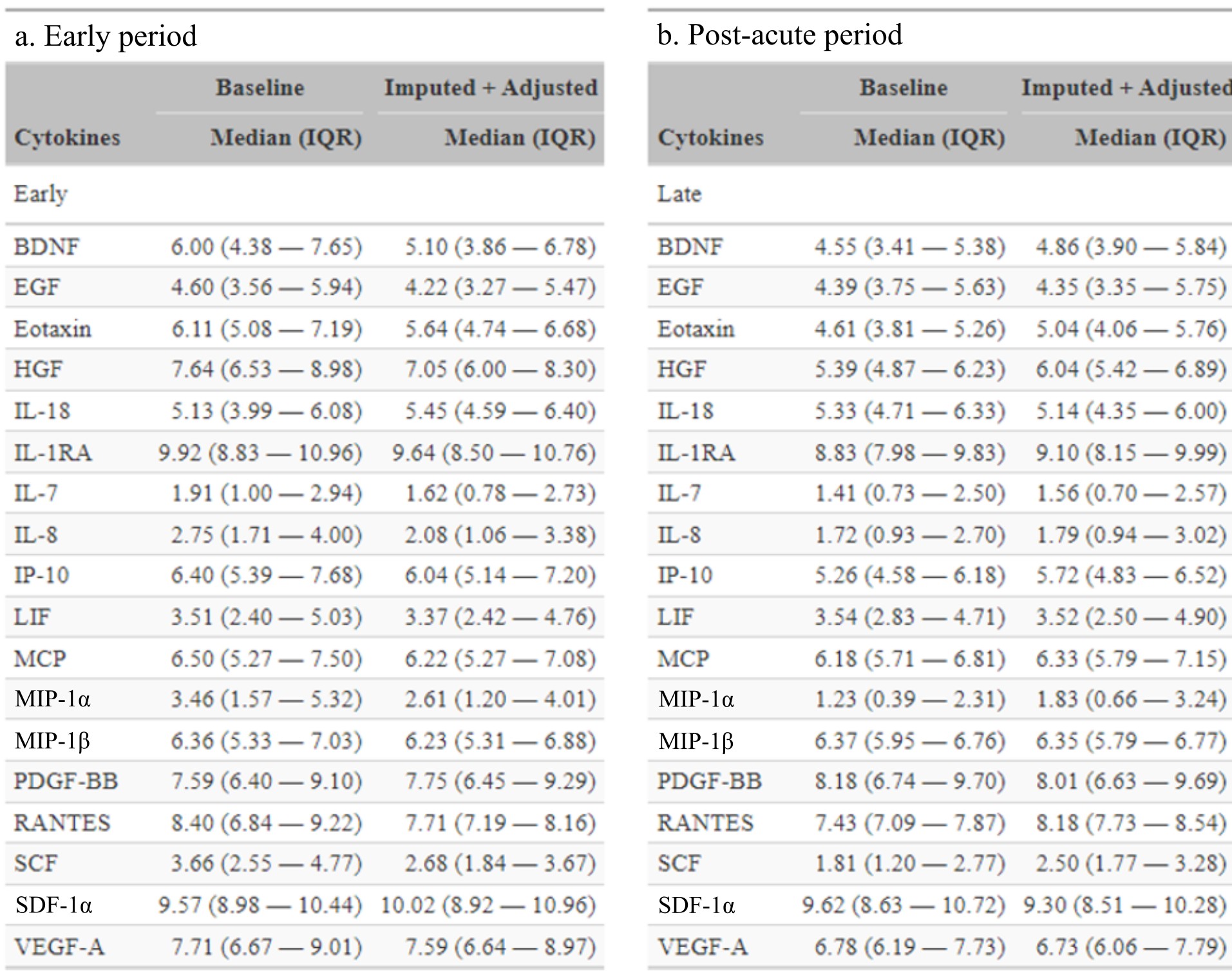


Supplemental Table 2. Summary of nominal p-values across multiple imputations for the Jonckheere-Terpstra trend tests of cytokine concentration between severity groups. Minimum, median, and maximum observed p-values across the 10 imputed datasets for each cytokine displayed along with the estimated most frequently observed direction of the trend. P-values below 0.05 noted with an asterisk.

|  | Early | | | | Post-acute | | | |
| --- | --- | --- | --- | --- | --- | --- | --- | --- |
| Cytokine | Min | Median | Max | Direction | Min | Median | Max | Direction |
| BDNF | 0.101 | 0.149 | 0.202 | Decreasing | 0.421 | 0.544 | 0.754 | Increasing |
| EGF | 0.544 | 0.854 | 0.99 | Increasing | 0.773 | 0.911 | 0.988 | Decreasing |
| Eotaxin | 0.724 | 0.733 | 0.756 | Increasing | 0.18 | 0.185 | 0.188 | Increasing |
| HGF | <0.001* | <0.001* | <0.001* | Increasing | 0.189 | 0.282 | 0.343 | Increasing |
| IL-18 | 0.001* | 0.002* | 0.003* | Increasing | 0.805 | 0.864 | 0.935 | Increasing |
| IL-1RA | 0.003* | 0.004* | 0.007* | Increasing | 0.01* | 0.012* | 0.016* | Increasing |
| IL-7 | 0.121 | 0.27 | 0.322 | Increasing | 0.442 | 0.607 | 0.81 | Increasing |
| IL-8 | 0.087 | 0.192 | 0.334 | Increasing | 0.027* | 0.034* | 0.055 | Increasing |
| IP-10 | <0.001* | <0.001* | <0.001* | Increasing | 0.057 | 0.063 | 0.068 | Increasing |
| LIF | 0.077 | 0.129 | 0.144 | Decreasing | 0.21 | 0.302 | 0.37 | Increasing |
| MCP-1 | 0.43 | 0.446 | 0.452 | Increasing | 0.697 | 0.705 | 0.715 | Decreasing |
| MIP-1α | 0.16 | 0.209 | 0.346 | Decreasing | <0.001* | <0.001* | <0.001* | Increasing |
| MIP-1β | 0.525 | 0.697 | 0.795 | Increasing | 0.035* | 0.062 | 0.068 | Increasing |
| PDGF-BB | 0.837 | 0.848 | 0.865 | Increasing | 0.322 | 0.328 | 0.334 | Increasing |
| RANTES | 0.03* | 0.31 | 0.773 | Decreasing | 0.392 | 0.421 | 0.442 | Decreasing |
| SCF | 0.424 | 0.557 | 0.699 | Increasing | 0.542 | 0.639 | 0.728 | Increasing |
| SDF-1α | 0.235 | 0.257 | 0.287 | Increasing | 0.257 | 0.289 | 0.346 | Increasing |
| VEGF-A | 0.001* | 0.002* | 0.002* | Increasing | 0.006* | 0.006* | 0.007* | Increasing |

Supplemental Table 3. Summary of nominal p-values across multiple imputations Wilcoxon signed rank tests of changes in cytokine concentration over time stratified by severity group. Minimum, median, and maximum observed p-values across the 10 imputed datasets for each cytokine displayed along with the estimated most frequently observed direction of the trend. P-values below 0.05 noted with an asterisk.

|  | Mild | | | | Moderate | | | | Severe | | | |
| --- | --- | --- | --- | --- | --- | --- | --- | --- | --- | --- | --- | --- |
| Cytokine | Min | Median | Max | Direction | Min | Median | Max | Direction | Min | Median | Max | Direction |
| BDNF | 0.056 | 0.081 | 0.169 | Decreased | 0.176 | 0.345 | 0.503 | Decreased | 0.148 | 0.148 | 0.148 | Decreased |
| EGF | <0.001* | 0.003* | 0.029* | Increased | 0.147 | 0.276 | 0.443 | Increased | 0.945 | 0.945 | 1 | Increased |
| Eotaxin | 0.021* | 0.023* | 0.023* | Decreased | 0.156 | 0.176 | 0.187 | Decreased | 0.148 | 0.148 | 0.148 | Decreased |
| HGF | 0.456 | 0.491 | 0.509 | Decreased | 0.042* | 0.054 | 0.113 | Decreased | 0.742 | 0.742 | 0.742 | Decreased |
| IL-18 | 0.768 | 0.779 | 0.79 | Decreased | 0.388 | 0.714 | 0.899 | Increased | 0.641 | 0.641 | 0.641 | Decreased |
| IL-1RA | 0.152 | 0.252 | 0.406 | Decreased | 0.424 | 0.644 | 1 | Decreased | 0.742 | 0.742 | 0.844 | Decreased |
| IL-7 | 0.089 | 0.246 | 0.406 | Decreased | 0.371 | 0.464 | 0.799 | Increased | 0.461 | 0.641 | 0.742 | Increased |
| IL-8 | 0.039* | 0.08 | 0.331 | Decreased | 0.013* | 0.05 | 0.305 | Increased | 0.383 | 0.895 | 1 | Decreased |
| IP-10 | 0.663 | 0.747 | 0.79 | Increased | 0.899 | 0.924 | 1 | Decreased | 0.039* | 0.039* | 0.055 | Decreased |
| LIF | 0.095 | 0.248 | 0.79 | Decreased | 0.503 | 0.799 | 0.949 | Decreased | 0.109 | 0.199 | 0.25 | Decreased |
| MCP-1 | 0.208 | 0.208 | 0.229 | Increased | 0.949 | 0.975 | 1 | Decreased | 0.547 | 0.547 | 0.547 | Decreased |
| MIP-1α | 0.001* | 0.004* | 0.039* | Decreased | 0.849 | 0.899 | 0.975 | Decreased | 0.547 | 0.547 | 0.547 | Decreased |
| MIP-1β | 0.004* | 0.006* | 0.015* | Increased | 0.098 | 0.182 | 0.222 | Increased | 0.461 | 0.461 | 0.461 | Increased |
| PDGF-BB | 0.833 | 0.966 | 1 | Decreased | 0.799 | 0.836 | 0.899 | Increased | 0.547 | 0.547 | 0.547 | Increased |
| RANTES | <0.001* | 0.002* | 0.01* | Increased | <0.001* | 0.006* | 0.016* | Increased | 0.109 | 0.195 | 0.547 | Increased |
| SCF | 0.034* | 0.111 | 0.277 | Increased | 0.388 | 0.473 | 0.503 | Increased | 0.461 | 0.461 | 0.461 | Increased |
| SDF-1α | 0.128 | 0.152 | 0.218 | Increased | 0.874 | 0.937 | 0.975 | Decreased | 0.844 | 0.945 | 0.945 | Decreased |
| VEGF-A | 0.303 | 0.36 | 0.39 | Decreased | 0.524 | 0.633 | 0.726 | Decreased | 0.742 | 0.742 | 0.742 | Decreased |

Supplemental Table 4. Summary of nominal p-values across multiple imputations for the Wilcoxon signed rank tests of changes in cytokine concentration over time stratified by impairment status and type of long-term symptom. Minimum, median, and maximum observed p-values across the 10 imputed datasets for each cytokine displayed along with the estimated most frequently observed direction of the trend. P-values below 0.05 noted with asterisk.

|  |  | Overall | | | | PROMIS Cognition | | | | PROMIS Fatigue | | | | mMRC Dyspnea | | | |
| --- | --- | --- | --- | --- | --- | --- | --- | --- | --- | --- | --- | --- | --- | --- | --- | --- | --- |
| Cytokine | Impaired? | Min | Median | Max | Direction | Min | Median | Max | Direction | Min | Median | Max | Direction | Min | Median | Max | Direction |
| BDNF | No | 0.565 | 0.626 | 0.727 | Decreased | 0.157 | 0.294 | 0.367 | Decreased | 0.024* | 0.032* | 0.054 | Decreased | 0.393 | 0.501 | 0.654 | Decreased |
| BDNF | Yes | 0.092 | 0.167 | 0.271 | Decreased | 0.084 | 0.146 | 0.322 | Decreased | 0.232 | 0.625 | 0.695 | Increased | 0.129 | 0.211 | 0.348 | Decreased |
| EGF | No | 0.011* | 0.044* | 0.367 | Increased | 0.008* | 0.056 | 0.166 | Increased | 0.007* | 0.031* | 0.116 | Increased | 0.007* | 0.035* | 0.221 | Increased |
| EGF | Yes | 0.109 | 0.191 | 0.44 | Increased | 0.064 | 0.118 | 0.375 | Increased | 0.193 | 0.254 | 0.557 | Increased | 0.202 | 0.307 | 0.413 | Increased |
| Eotaxin | No | 0.067 | 0.075 | 0.08 | Decreased | 0.003* | 0.003* | 0.004* | Decreased | 0.002* | 0.002* | 0.002* | Decreased | 0.02* | 0.024* | 0.026* | Decreased |
| Eotaxin | Yes | 0.005* | 0.005* | 0.006* | Decreased | 0.193 | 0.193 | 0.193 | Decreased | 0.322 | 0.322 | 0.322 | Decreased | 0.015* | 0.016* | 0.019* | Decreased |
| HGF | No | 0.635 | 0.653 | 0.708 | Decreased | 0.082 | 0.101 | 0.142 | Decreased | 0.015* | 0.022* | 0.031* | Decreased | 0.347 | 0.369 | 0.405 | Decreased |
| HGF | Yes | 0.02* | 0.029* | 0.045* | Decreased | 0.322 | 0.349 | 0.492 | Decreased | 0.375 | 0.375 | 0.375 | Increased | 0.055 | 0.077 | 0.117 | Decreased |
| IL-18 | No | 0.499 | 0.6 | 0.881 | Increased | 0.6 | 0.827 | 0.907 | Decreased | 0.803 | 0.871 | 0.996 | Increased | 0.256 | 0.315 | 0.536 | Increased |
| IL-18 | Yes | 0.935 | 0.984 | 1 | Decreased | 0.193 | 0.193 | 0.193 | Increased | 0.492 | 0.492 | 0.492 | Increased | 0.562 | 0.611 | 0.662 | Decreased |
| IL-1RA | No | 0.881 | 0.901 | 0.98 | Decreased | 0.247 | 0.32 | 0.43 | Decreased | 0.096 | 0.111 | 0.176 | Decreased | 0.417 | 0.529 | 0.717 | Decreased |
| IL-1RA | Yes | 0.08 | 0.096 | 0.152 | Decreased | 0.492 | 0.557 | 0.846 | Decreased | 0.375 | 0.557 | 0.695 | Increased | 0.202 | 0.224 | 0.324 | Decreased |
| IL-7 | No | 0.708 | 0.911 | 0.98 | Increased | 0.389 | 0.703 | 0.851 | Decreased | 0.811 | 0.899 | 0.996 | Increased | 0.468 | 0.75 | 0.983 | Increased |
| IL-7 | Yes | 0.465 | 0.693 | 1 | Increased | 0.02* | 0.037* | 0.131 | Increased | 0.232 | 0.349 | 0.77 | Increased | 0.714 | 0.943 | 1 | Decreased |
| IL-8 | No | 0.075 | 0.337 | 0.901 | Increased | 0.306 | 0.647 | 0.907 | Increased | 0.243 | 0.799 | 0.988 | Increased | 0.221 | 0.536 | 0.848 | Increased |
| IL-8 | Yes | 0.237 | 0.584 | 0.968 | Decreased | 0.492 | 0.77 | 1 | Increased | 0.16 | 0.462 | 0.922 | Increased | 0.455 | 0.841 | 1 | Decreased |
| IP-10 | No | 0.6 | 0.717 | 0.803 | Increased | 0.78 | 0.823 | 0.915 | Decreased | 0.31 | 0.338 | 0.436 | Decreased | 0.815 | 0.966 | 1 | Increased |
| IP-10 | Yes | 0.158 | 0.191 | 0.245 | Decreased | 0.193 | 0.232 | 0.322 | Decreased | 0.492 | 0.557 | 0.695 | Increased | 0.229 | 0.269 | 0.361 | Decreased |
| LIF | No | 0.199 | 0.303 | 0.565 | Decreased | 0.028* | 0.094 | 0.2 | Decreased | 0.098 | 0.147 | 0.461 | Decreased | 0.156 | 0.252 | 0.609 | Decreased |
| LIF | Yes | 0.36 | 0.605 | 0.715 | Decreased | 0.275 | 0.375 | 0.922 | Increased | 0.77 | 0.922 | 1 | Increased | 0.485 | 0.645 | 0.859 | Decreased |
| MCP-1 | No | 0.129 | 0.146 | 0.157 | Increased | 0.123 | 0.131 | 0.145 | Increased | 0.273 | 0.289 | 0.32 | Increased | 0.256 | 0.279 | 0.294 | Increased |
| MCP-1 | Yes | 0.7 | 0.777 | 0.855 | Increased | 0.922 | 1 | 1 | Decreased | 0.432 | 0.557 | 0.557 | Increased | 0.485 | 0.53 | 0.611 | Increased |
| MIP-1α | No | 0.515 | 0.662 | 0.96 | Decreased | 0.064 | 0.111 | 0.296 | Decreased | 0.024* | 0.043* | 0.179 | Decreased | 0.369 | 0.523 | 0.983 | Decreased |
| MIP-1α | Yes | 0.021* | 0.034* | 0.061 | Decreased | 0.322 | 0.432 | 0.625 | Decreased | 0.625 | 0.77 | 0.846 | Increased | 0.034* | 0.051 | 0.062 | Decreased |
| MIP-1β | No | <0.001* | 0.003* | 0.005* | Increased | 0.002* | 0.005* | 0.008* | Increased | 0.007* | 0.013* | 0.032* | Increased | 0.004* | 0.009* | 0.017* | Increased |
| MIP-1β | Yes | 0.36 | 0.382 | 0.529 | Increased | 0.375 | 0.375 | 0.695 | Increased | 0.064 | 0.064 | 0.064 | Increased | 0.194 | 0.211 | 0.313 | Increased |
| PDGF-BB | No | 0.881 | 0.881 | 0.92 | Increased | 0.552 | 0.64 | 0.726 | Increased | 0.651 | 0.764 | 0.867 | Increased | 0.766 | 0.807 | 0.848 | Increased |
| PDGF-BB | Yes | 0.213 | 0.271 | 0.318 | Increased | 0.049* | 0.064 | 0.084 | Increased | 0.16 | 0.16 | 0.16 | Increased | 0.239 | 0.269 | 0.324 | Increased |
| RANTES | No | <0.001* | <0.001* | 0.003* | Increased | <0.001* | <0.001* | <0.001* | Increased | <0.001* | <0.001* | 0.003* | Increased | <0.001* | 0.002* | 0.01* | Increased |
| RANTES | Yes | 0.019* | 0.064 | 0.221 | Increased | 0.064 | 0.277 | 0.846 | Increased | 0.014* | 0.037* | 0.16 | Increased | 0.004* | 0.021* | 0.148 | Increased |
| SCF | No | 0.38 | 0.549 | 1 | Increased | 0.196 | 0.323 | 0.696 | Increased | 0.384 | 0.499 | 0.835 | Increased | 0.468 | 0.662 | 0.966 | Increased |
| SCF | Yes | 0.271 | 0.564 | 0.871 | Increased | 0.557 | 0.695 | 0.77 | Increased | 0.049* | 0.322 | 0.922 | Increased | 0.194 | 0.456 | 0.768 | Increased |
| SDF-1α | No | 0.269 | 0.291 | 0.34 | Increased | 0.579 | 0.677 | 0.772 | Increased | 0.572 | 0.658 | 0.764 | Increased | 0.565 | 0.609 | 0.67 | Increased |
| SDF-1α | Yes | 0.584 | 0.626 | 0.761 | Decreased | 0.695 | 0.77 | 0.922 | Increased | 0.557 | 0.557 | 0.695 | Increased | 0.897 | 0.962 | 1 | Decreased |
| VEGF-A | No | 0.803 | 0.891 | 0.94 | Decreased | 0.467 | 0.525 | 0.559 | Decreased | 0.142 | 0.176 | 0.207 | Decreased | 0.815 | 0.915 | 1 | Decreased |
| VEGF-A | Yes | 0.213 | 0.267 | 0.299 | Decreased | 0.492 | 0.557 | 0.625 | Decreased | 0.275 | 0.299 | 0.322 | Increased | 0.185 | 0.234 | 0.269 | Decreased |

Supplemental Table 5. Table of percent of individuals with 0-3 long-term symptoms (Total). Presents the percent of each group with a particular long-term symptom, e.g. among the 24% of people with one long-term symptom, 76% of them had dyspnea.

| Number of Long-term Symptoms | Dyspnea | Impaired Cognition | Fatigue | Total |
| --- | --- | --- | --- | --- |
| 0 | 0 | 0 | 0 | 0.42 |
| 1 | 0.76 | 0.11 | 0.13 | 0.24 |
| 2 | 0.84 | 0.5 | 0.66 | 0.15 |
| 3 | 1 | 1 | 1 | 0.09 |
| Unknown | 0.18 | 0.11 | 0.25 | 0.11 |


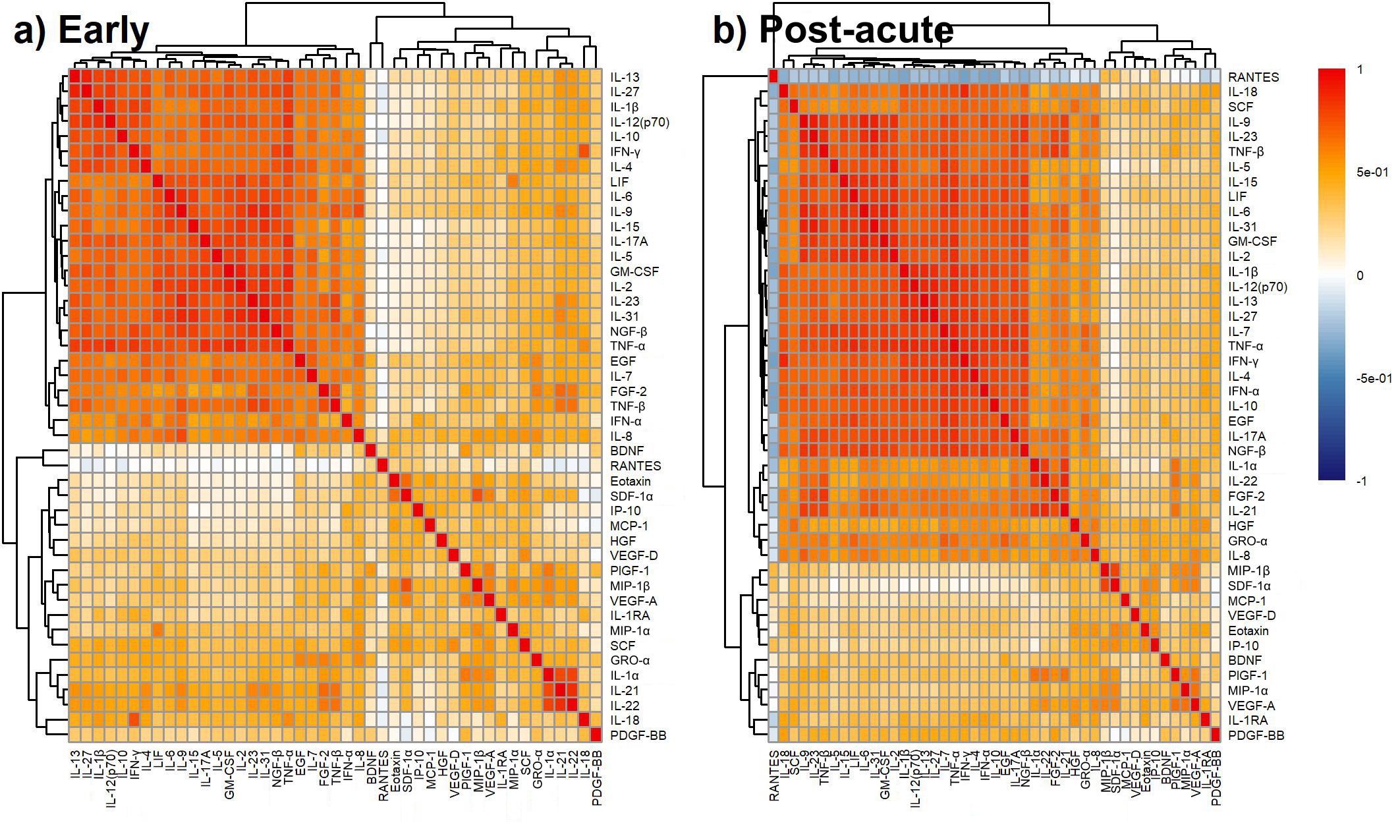


Supplemental Figure 1. Correlation plot of all 45 cytokines in the multiplex assay after multiple imputation and batch effect correction in early (a) and post-acute (b) sampling periods. Point estimate of correlations pooled by transforming to Fisher Z scores, averaging, and then transforming back to correlations.

Supplemental Table 6. Results of the Jonckheere Terpstra trend test with imputed severity values included in the model. Only cytokines with at least one nominal p-value below 0.1 displayed. Q-values below 0.05 noted with an asterisk.

|  | Early period | | Post-acute period | |
| --- | --- | --- | --- | --- |
| Cytokine | q-value | Direction | q-value | Direction |
| HGF | <0.001* | Increasing | 0.669 | Increasing |
| IL-1RA | 0.01* | Increasing | 0.152 | Increasing |
| IL-8 | 0.298 | Increasing | 0.214 | Increasing |
| IL-18 | 0.005* | Increasing | 0.813 | Increasing |
| IP-10 | <0.001* | Increasing | 0.541 | Increasing |
| MIP-1α | 0.588 | Decreasing | 0.004* | Increasing |
| VEGF-A | 0.001* | Increasing | 0.09 | Increasing |

Supplemental Figure 2. Forest plots of the age, sex, and CCI adjusted logistic regression of pair-wise COVID-19 severity comparisons using imputed severity values in the model. Only cytokines with at least one nominal p-value below 0.1 displayed. Benjamini-Hochberg q-values displayed next to their respective estimates.


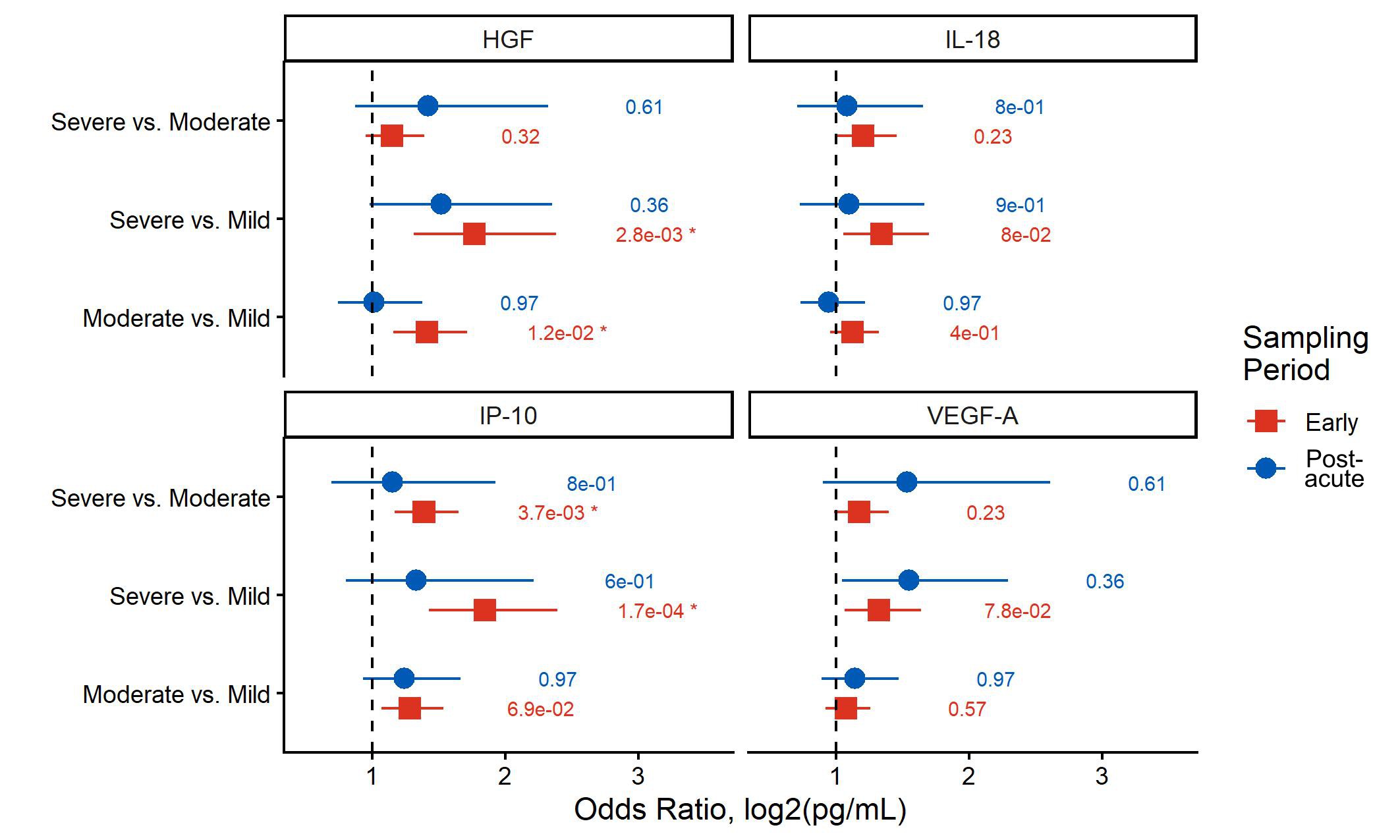


Supplemental Table 7. Results of the Jonckheere Terpstra trend test on the subset of participants with only positive PCR or antigen tests around enrollment. Only cytokines with at least one nominal p-value below 0.1 displayed. Q-values below 0.05 noted with an asterisk.

|  | Early period | | Post-acute period | |
| --- | --- | --- | --- | --- |
| Cytokine | q-value | Direction | q-value | Direction |
| HGF | <0.001* | Increasing | 0.579 | Increasing |
| IL-1RA | 0.002* | Increasing | 0.021* | Increasing |
| IL-7 | 0.122 | Increasing | 0.335 | Increasing |
| IL-8 | 0.001* | Increasing | 0.328 | Increasing |
| IL-18 | 0.004* | Increasing | 0.626 | Increasing |
| IP-10 | <0.001* | Increasing | 0.328 | Increasing |
| MCP-1 | 0.029* | Increasing | 0.754 | Decreasing |
| MIP-1α | 0.059 | Increasing | 0.002* | Increasing |
| SCF | 0.004* | Increasing | 0.626 | Increasing |
| SDF-1α | 0.064 | Increasing | 0.664 | Increasing |
| VEGF-A | 0.002* | Increasing | 0.07 | Increasing |

Supplemental Figure 3. Forest plots of age, sex, CCI adjusted logistic regression on pairwise odds of more severe disease in the subset of participants with only positive SARS-CoV-2 PCR or antigen tests. Only cytokines with at least one nominal p-value below 0.1 displayed. Estimates with a Benjamini-Hochberg q-value below 0.05 noted with an asterisk.


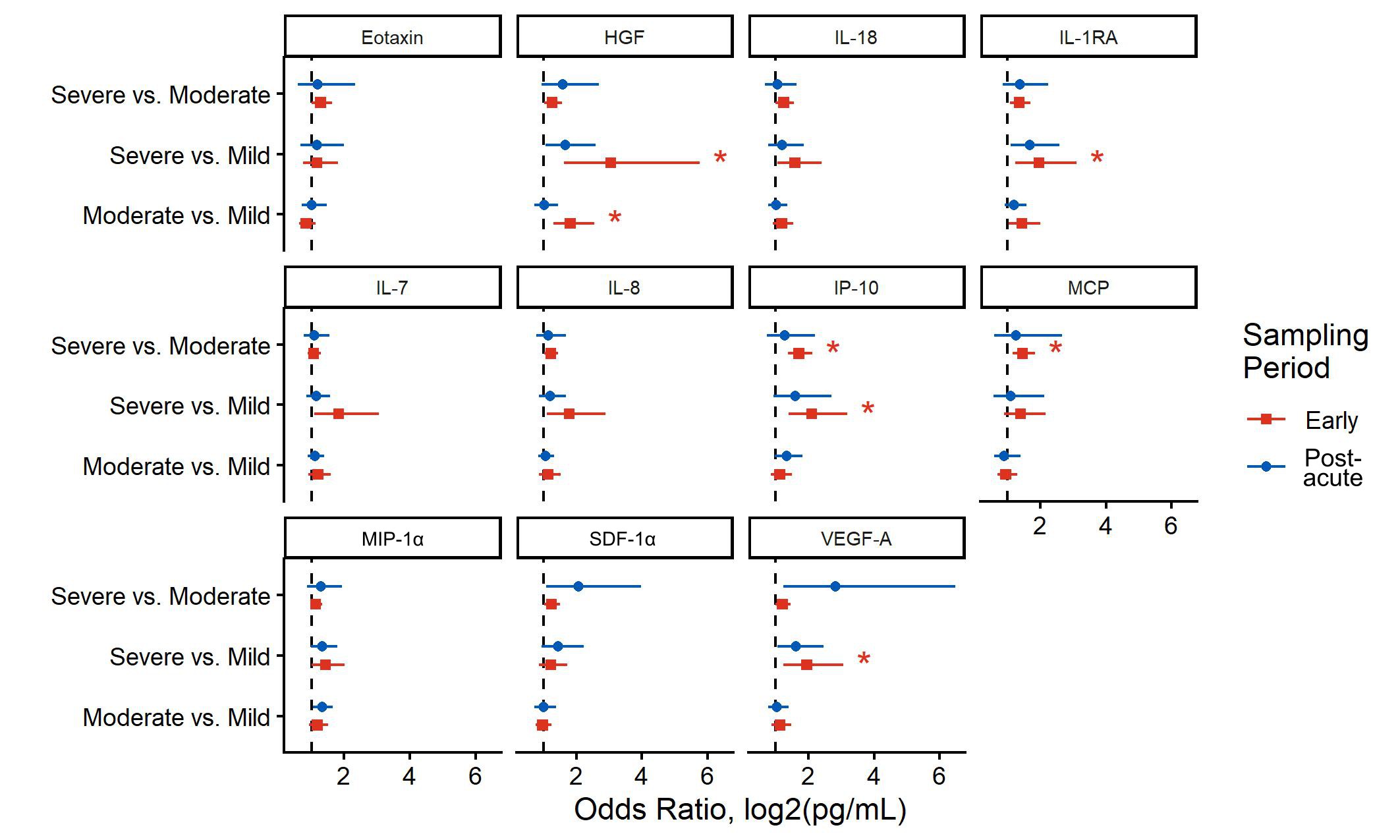


Supplemental Figure 4. Forest plots of age, sex, and CCI adjusted logistic regression of pair-wise COVID comparisons with imputed severity values included in the model. Benjamini-Hochberg q-values displayed next to their respective estimates.


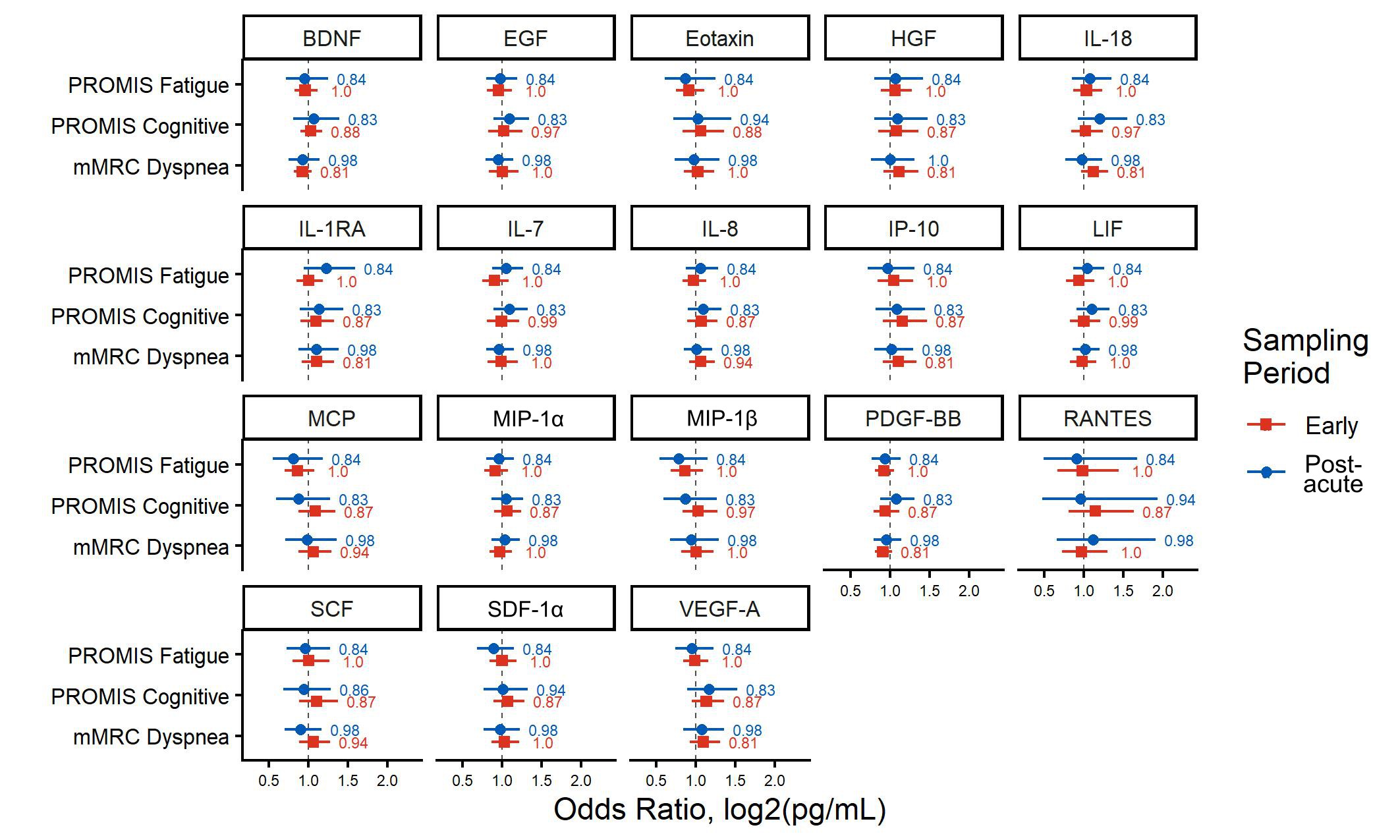


Supplemental Figure 5. Forest plots of age, sex, and CCI adjusted logistic regression on odds of impairment in long-term symptoms in the subset of participants with strictly positive SARS-CoV-2 PCR or antigen tests. Benjamini-Hochberg q-values displayed next to their respective estimates.


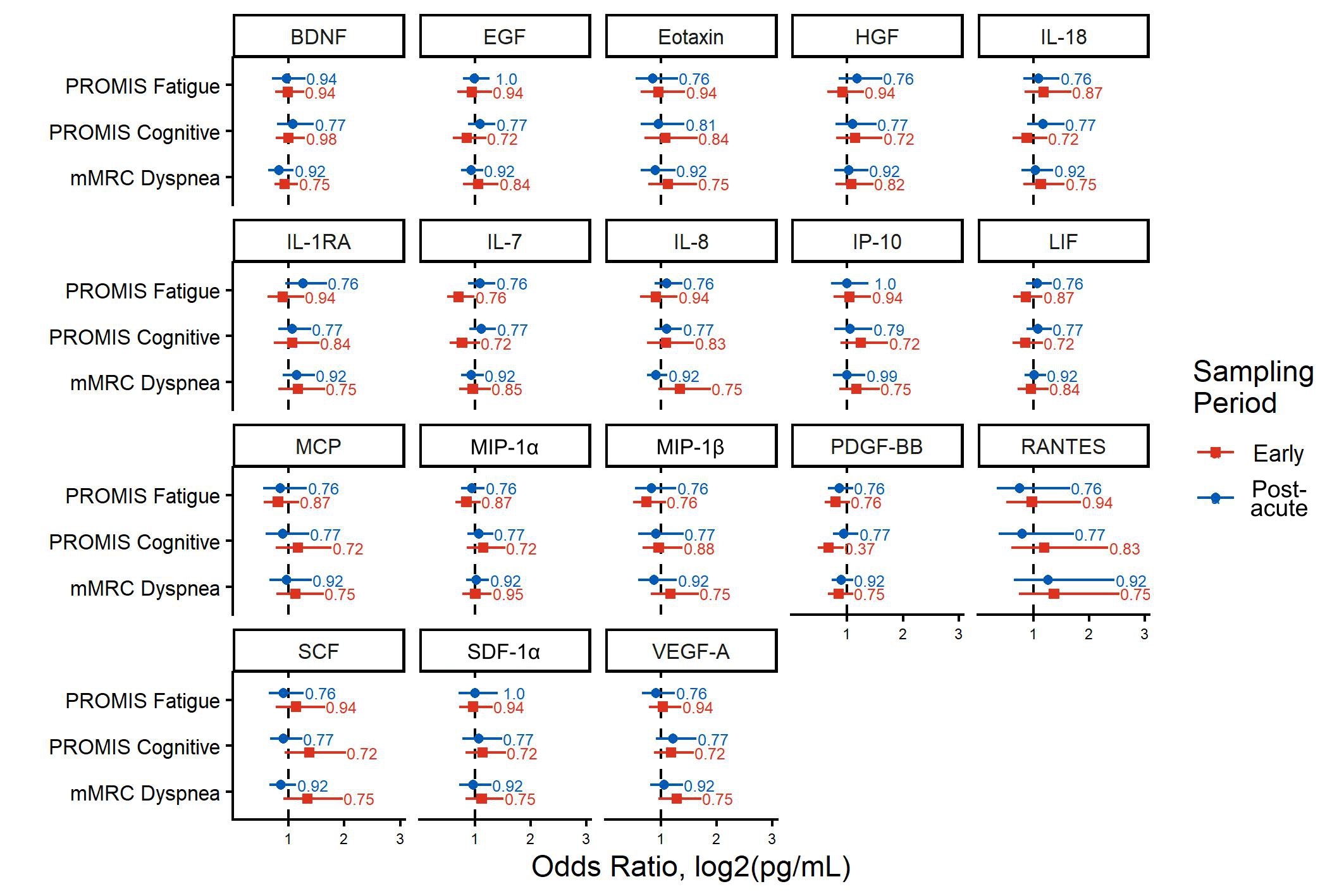


Supplemental Table 8. Results of Wilcoxon Signed Rank tests on paired early and post-acute period samples with imputed COVID-19 severity included. Average imputed group size: Mild N = 33, Moderate N = 29, Severe N = 10. P-values and q-values below 0.05 noted with an asterisk.

| Cytokine | Severity | Nominal p-value | BH adjusted q-value | Direction |
| --- | --- | --- | --- | --- |
| BDNF | Mild | 0.082 | 0.247 | Decreased |
| EGF | Mild | 0.002* | 0.009* | Increased |
| Eotaxin | Mild | 0.001* | 0.009* | Decreased |
| Eotaxin | Moderate | 0.016* | 0.096 | Decreased |
| Eotaxin | Severe | 0.088 | 0.591 | Decreased |
| HGF | Moderate | 0.006* | 0.056 | Decreased |
| IL-8 | Moderate | 0.034* | 0.153 | Increased |
| IP-10 | Severe | 0.024* | 0.436 | Decreased |
| MIP-1α | Mild | 0.003* | 0.011* | Decreased |
| MIP-1β | Mild | 0.002* | 0.01* | Increased |
| RANTES | Mild | <0.001* | 0.002* | Increased |
| RANTES | Moderate | 0.004* | 0.056 | Increased |

Supplemental Table 9. Results of Wilcoxon Signed Rank tests of each severity group using the subset of participants that tested positive for SARS-CoV-2 infections via PCR or antigen tests. Mild N = 10, Moderate N = 18, Severe N = 8.  P-values and q-values below 0.05 noted with an asterisk.

| Cytokine | Severity | Nominal p-value | BH adjusted q-value | Direction |
| --- | --- | --- | --- | --- |
| EGF | Mild | 0.01* | 0.178 | Increased |
| HGF | Moderate | 0.048* | 0.434 | Decreased |
| IP-10 | Severe | 0.046* | 0.814 | Decreased |
| MIP-1β | Mild | 0.039* | 0.339 | Increased |
| RANTES | Mild | 0.056 | 0.339 | Increased |
| RANTES | Moderate | 0.007* | 0.126 | Increased |
| SDF-1α | Mild | 0.082 | 0.37 | Increased |

Supplemental Table 10. Results of Wilcoxon Signed Rank tests on paired early and post-acute period samples using imputed long-term symptom status. Average imputed group size: Cognition – Normal/Impaired N = 58/14; Fatigue – Normal/Impaired N = 58/14; Dyspnea – Normal/Impaired N = 37/35; Overall – Normal/Impaired N = 31/41. P-values and q-values below 0.05 noted with an asterisk.

| Cytokine | Measure | Impaired? | Nominal p-value | BH adjusted p-value | Direction |
| --- | --- | --- | --- | --- | --- |
| BDNF | Overall | Yes | 0.009* | 0.041* | Decreased |
| BDNF | PROMIS Fatigue | No | 0.001* | 0.017* | Decreased |
| EGF | mMRC Dyspnea | No | 0.003* | 0.02* | Increased |
| EGF | Overall | No | 0.005* | 0.028* | Increased |
| EGF | PROMIS Fatigue | No | 0.007* | 0.037* | Increased |
| Eotaxin | mMRC Dyspnea | No | 0.003* | 0.02* | Decreased |
| Eotaxin | mMRC Dyspnea | Yes | 0.002* | 0.02* | Decreased |
| Eotaxin | Overall | Yes | <0.001* | <0.001* | Decreased |
| Eotaxin | PROMIS Cognition | No | <0.001* | 0.002* | Decreased |
| Eotaxin | PROMIS Fatigue | No | <0.001* | 0.002* | Decreased |
| HGF | Overall | Yes | 0.005* | 0.028* | Decreased |
| HGF | PROMIS Fatigue | No | 0.002* | 0.018* | Decreased |
| MIP-1α | mMRC Dyspnea | Yes | 0.014* | 0.069 | Decreased |
| MIP-1α | Overall | Yes | 0.005* | 0.028* | Decreased |
| MIP-1α | PROMIS Fatigue | No | 0.006* | 0.034* | Decreased |
| MIP-1β | mMRC Dyspnea | No | 0.002* | 0.02* | Increased |
| MIP-1β | Overall | No | <0.001* | 0.001* | Increased |
| MIP-1β | PROMIS Cognition | No | 0.002* | 0.02* | Increased |
| MIP-1β | PROMIS Fatigue | No | 0.012* | 0.053 | Increased |
| RANTES | mMRC Dyspnea | No | <0.001* | <0.001* | Increased |
| RANTES | mMRC Dyspnea | Yes | 0.002* | 0.02* | Increased |
| RANTES | Overall | No | <0.001* | <0.001* | Increased |
| RANTES | Overall | Yes | 0.005* | 0.028* | Increased |
| RANTES | PROMIS Cognition | No | <0.001* | <0.001* | Increased |
| RANTES | PROMIS Fatigue | No | <0.001* | <0.001* | Increased |
| RANTES | PROMIS Fatigue | Yes | 0.004* | 0.027* | Increased |

Supplemental Table 11. Results of Wilcoxon Signed rank tests for individual long-term symptom groups in the subset of participants with strictly positive PCR and antigen tests. Cognition – Normal, Impaired N = 23/6; Fatigue – Normal, Impaired N = 24/5; Dyspnea – Normal, Impaired N = 13/14; Overall – Normal, Impaired N = 12/15. P-values and q-values below 0.05 noted with an asterisk.

| Cytokine | Measure | Impaired? | Nominal p-value | BH adjusted q-value | Direction |
| --- | --- | --- | --- | --- | --- |
| RANTES | Overall | No | 0.003* | 0.122 | Increased |
| RANTES | PROMIS Cognition | No | <0.001* | 0.004* | Increased |
| RANTES | PROMIS Fatigue | No | 0.003* | 0.112 | Increased |
